# *Ehd4* Encodes a Novel and *Oryza*-Genus-Specific Regulator of Photoperiodic Flowering in Rice

**DOI:** 10.1371/journal.pgen.1003281

**Published:** 2013-02-21

**Authors:** He Gao, Xiao-Ming Zheng, Guilin Fei, Jun Chen, Mingna Jin, Yulong Ren, Weixun Wu, Kunneng Zhou, Peike Sheng, Feng Zhou, Ling Jiang, Jie Wang, Xin Zhang, Xiuping Guo, Jiu-Lin Wang, Zhijun Cheng, Chuanyin Wu, Haiyang Wang, Jian-Min Wan

**Affiliations:** 1 National Key Laboratory for Crop Genetics and Germplasm Enhancement, Jiangsu Plant Gene Engineering Research Center, Nanjing Agricultural University, Nanjing, China; 2 National Key Facility for Crop Gene Resources and Genetic Improvement, Institute of Crop Science, Chinese Academy of Agricultural Sciences, Beijing, China; Peking University, China

## Abstract

Land plants have evolved increasingly complex regulatory modes of their flowering time (or heading date in crops). Rice (*Oryza sativa* L.) is a short-day plant that flowers more rapidly in short-day but delays under long-day conditions. Previous studies have shown that the *CO*-*FT* module initially identified in long-day plants (Arabidopsis) is evolutionary conserved in short-day plants (*Hd1*-*Hd3a* in rice). However, in rice, there is a unique *Ehd1*-dependent flowering pathway that is *Hd1*-independent. Here, we report isolation and characterization of a positive regulator of *Ehd1*, *Early heading date 4* (*Ehd4*). *ehd4* mutants showed a never flowering phenotype under natural long-day conditions. Map-based cloning revealed that *Ehd4* encodes a novel CCCH-type zinc finger protein, which is localized to the nucleus and is able to bind to nucleic acids *in vitro* and transactivate transcription in yeast, suggesting that it likely functions as a transcriptional regulator. *Ehd4* expression is most active in young leaves with a diurnal expression pattern similar to that of *Ehd1* under both short-day and long-day conditions. We show that *Ehd4* up-regulates the expression of the “florigen” genes *Hd3a* and *RFT1* through *Ehd1,* but it acts independently of other known *Ehd1* regulators. Strikingly, *Ehd4* is highly conserved in the *Oryza* genus including wild and cultivated rice, but has no homologs in other species, suggesting that *Ehd4* is originated along with the diversification of the *Oryza* genus from the grass family during evolution. We conclude that *Ehd4* is a novel *Oryza*-genus-specific regulator of *Ehd1*, and it plays an essential role in photoperiodic control of flowering time in rice.

## Author Summary

Rice is an important source of calories for mankind. Flowering time determines cropping seasons and regional adaptability of crops. Rice is originated from its wild progenitor, *O*. *rufipogon*, which is mainly distributed at tropical latitudes with a northern limit about 28 °N, more than 10,000 years ago. However, cultivated rice is now grown widely in Asia, with a northern limit of nearly 53 °N. The northward expansion of cultivated rice must be accompanied by human selection of the flowering time trait during domestication and breeding, to secure a harvest before cold weather approaches. By identifying a rice mutant that never flowers under natural long-day conditions (NLDs), we cloned *Ehd4* as a novel transcriptional regulator that promotes flowering through activation of two “florigen” genes, the signals for flowering initiation. We found that *Ehd4* has two major haplotypes: Hap_2 is the major haplotype in *indica* accessions mostly distributed in lower latitude and elevation zones, whereas Hap_3 is the major haplotype in *japonica* accessions mostly distributed in higher latitudes and elevation zones. Genetic studies showed that Hap_3 is functionally more potent in promoting flowering under NLDs, implying that *Ehd4* may have contributed to the northward expansion and regional adaptability of cultivated rice into higher latitudes.

## Introduction

Flowering is a profound transition from vegetative to reproductive development in plants, and is largely determined by genetic pathways that integrate endogenous and environmental signals [1]. Plants control flowering by perceiving their surroundings, such as day-length (photoperiod) and temperature that is synchronized with seasonal changes, in order to maximize their reproductive fitness [2]. Flowering time or heading date in crops is also a critical agronomic trait that determines the cropping season and regional adaptability of plants. Thus, control of flowering time has been extensively studied by plant breeders and scientists for more than 100 years [3].

Photoperiod control of flowering refers to the ability of plants to measure day-length and use it as an indicator to initiate flowering. Extensive studies in a model long-day plant (LDP), *Arabidopsis thaliana*, have revealed that light regulation of the *GIGANTEA* (*GI*)-*CONSTANT* (*CO*)-*FLOWERING LOCUS T* (*FT*) pathway is essential for integrating cellular signals from light signaling transduction and the circadian clock to promote flowering under long-day conditions (LDs) [4]–[6]. Phytochrome A (phyA), phytochrome B (phyB) and cryptochrome 2 (cry2) regulate *FT* expression by post-translationally regulating CO protein [7], [8]. In addition, blue light promotes *CO* expression by stabilizing the FLAVIN-binding KELCH DOMAIN F BOX PROTEIN1 (FKF1)-GI protein complex [9], [10]. CO, a zinc finger transcription factor, promotes *FT* expression under LDs by directly binding to its promoter [11], [12]. FT, a small mobile protein functioning as the ‘florigen’, is synthesized in the phloem of leaves, and is then transported to the apical meristem where it initiates flowering by inducing the expression of the floral meristem identity genes, such as *AP1* [13]–[15].

Rice (*Oryza sativa* L.) is an important source of calories for mankind and a model short-day plant (SDP) that flowers more rapidly in short-day conditions (SDs) but delays under LDs with a critical day-length response [16], [17]. Previous studies have revealed that rice flowering is regulated both by a “SD-activation pathway” and a “LD-suppression pathway”. *OsGIGANTEA* (*OsGI*), *Heading date 1* (*Hd1*) and *Heading date 3a* (*Hd3a*) have been identified as the counterpart of *GI*, *CO* and *FT*, respectively [18]–[20]. *Hd1* executes dual function that promotes flowering by regulating *Hd3a* (the major SD ‘florigen’) expression under SDs, but suppresses it through unknown mechanisms under LDs [19], [21], [22]. However, the *OsGI*-*Hd1*-*Hd3a* pathway only plays a limited role in flowering time control in rice because there is a high degree of polymorphism in *Hd1* and non-functional alleles of *Hd1* are associated with only moderate phenotypic changes [23].

Rice has a unique, *Hd1*-independent flowering pathway that is mediated by *Early heading date 1* (*Ehd1*). *Ehd1* encodes a B-type response regulator that is highly conserved in cultivated rice, but has no homolog in Arabidopsis [23], [24]. It has been shown that *Ehd1* positively regulates the expression of *Hd3a* and *RICE FLOWERING LOCUS T 1* (*RFT1*), the closest paralog of *Hd3a* that works as a LD ‘florigen’ 22,24,25. Circumstantial evidence suggests that *Ehd1* is a critical convergence point of regulation by multiple signaling pathways. Among them, *OsphyB* inhibits flowering under both SDs and LDs by suppressing *Hd3a* expression through posttranslational modification of HD1 protein function and transcriptional suppression of *Ehd1* expression [25]–[27]. The *OsphyB*-mediated suppression of *Ehd1* is regulated by *OsCOL4*, which encodes a protein containing two B-box zinc finger domains and one CCT domain and it also acts as a constitutive suppressor of flowering in rice under both SD and LD conditions [26], [27]. In addition, both *Ghd7* (*Grain number*, *plant height and heading date 7*), encoding a CCT domain protein [28], and *DTH8* (*Days to heading 8*), encoding a putative HAP3 subunit of the CCAAT-box-binding transcription factor, down-regulate *Ehd1* expression and delay flowering under LDs [29]. On the other hand, *Ehd1* expression is promoted by a number of positive regulators. Among them, *OsMADS51* encodes a type I MADS-box protein and induces *Ehd1* expression under SDs [30], whereas a rice homolog of Arabidopsis *SOC1* (*Suppressor of Overexpression of Constant1*), *OsMADS50*, was identified as a promoter of *Ehd1* expression under LDs [31]. Recently, it was shown that *Ehd1* expression could be independently up-regulated by *Early heading date 2/Rice Indeterminate 1*/*Oryza sativa Indeterminate 1* (referred to as *Ehd2* hereafter) and *Early heading date 3* (*Ehd3*) under both SDs and LDs [32]–[34]. The former encodes a Cys2/His2-type zinc finger protein with high homology to maize *Indeterminate1* [35], while the latter encodes a putative plant homeodomain (PHD) finger-containing protein. Notably, loss of function of *OsMADS50*, *Ehd2* and *Ehd3* showed a never-flowering phenotype under LDs [25], [32], [34], [36]. Thus, it appears that *OsMAD50*, *Ehd2, Ehd3* and *Ehd1* may constitute a “LD-activation pathway” in rice. Although these studies have revealed much insight into the photoperiodic flowering of rice, the underlying molecular mechanisms are still not well understood.

Here we report the identification of *Early heading date 4* (*Ehd4*) using a mutagenesis approach and its positional cloning. *Ehd4* encodes a novel CCCH (C-X_7_-C-X_5_-C-X_3_-H)-type zinc finger protein and it acts as a critical regulator promoting flowering under both SDs and LDs, particularly under LDs. Mutation in *Ehd4* causes a never-flowering phenotype under natural long-day conditions (NLDs). EHD4 protein is localized to the nucleus and it has nucleic acid-binding and transcriptional activation properties, consistent with a plausible function as a transcription factor. We show that *Ehd4* promotes flowering by up-regulating the expression of *Hd3a* and *RFT1* through stimulation of *Ehd1* expression. Interestingly, *Ehd4* is highly conserved in the *Oryza* genus and it has no homologs in other plant species. Thus, our findings identified a novel, highly conserved rice-specific regulator of flowering time.

## Results

### Characterization of the late flowering mutant *ehd4*

**Figure 1:**
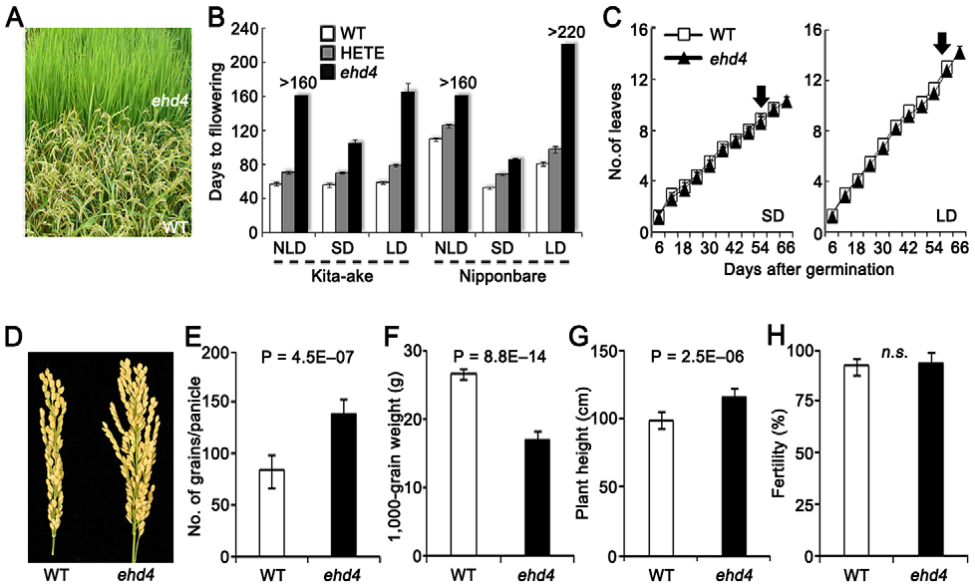
Characterization of *Ehd4*. (*A*) Never-flowering phenotype of *ehd4* mutants in field (*Top*). WT, Kita-ake wild-type plants (*Bottom*). (*B*) Flowering time of *ehd4*, heterozygote (HETE) and WT plants under different day length conditions in Kita-ake (day-length neutral) and Nipponbare (day-length sensitive) backgrounds (*n* = 12). ND, natural-day; SD, short-day; LD, long-day. (*C*) *ehd4* plants had the same leaf emergence rate as WT (Kita-ake) under both SDs and LDs (*n* = 8). Arrow indicates the flowering time of WT plants. (*D*) Panicle morphology of WT and *ehd4* plants. (E) to (H) Comparisons of grain number per panicle (*E*), 1000-grain weight (*F*), plant height (*G*) and fertility (*H*) between WT and *ehd4* plants. Values are means±s.d. (standard deviations) (*n* = 15). **Significant at 1% level; n.s., not significant.

In an effort to isolate genes that are essential for promoting flowering time in rice, we generated a large T-DNA population in a day-length neutral, early flowering variety Kita-ake (*O. sativa* ssp. *japonica*). Kita-ake (Kit) has been widely used in rice transformation experiments because of its short life cycle. Kit flowers about two months after germination under both SDs (10 h light/14 h dark) and LDs (14.5 h light/9.5 h dark) conditions in the controlled growth chamber, as well as under natural long-day field conditions (NLDs) in Beijing (39°54′N, 116°23′E), North China (Figure 1*A* and 1*B*). To understand the day-length neutral nature of Kita-ake, we cloned ten genes reported to have significant effect on flowering time in rice, including seven genes that promote flowering (*Ehd 1* to *3*, *OsMADS50*, *OsMADS51*, *Hd3a* and *RFT1*) and three genes that suppress flowering under LDs (*Hd6*, *Hd1* and *Ghd7*), and compared them with the corresponding genes in Nipponbare (Nip), a *japonica* variety that is sensitive to day-length. Those flowering-promoting genes are identical in Kit and Nip varieties, except *OsMADS51* that contains one amino acid variation (Figure S1). In contrast, Kit has an immature stop in *Ghd7* and a 36-bp insertion and two amino acid changes in *Hd1* (Figure S1). Although there is no difference in *Hd6* sequences between Kit and Nip, both of them have an early stop compared with the allele of the *indica* variety Kasalash (Figure S1), which delays flowering in Nip background under LDs [37], [38]. Therefore, complete or partial loss of function of those three genes in Kit could at least partially explain its insensitivity to day-length.

We screened our T-DNA population in the NLDs and identified a mutant that failed to flower during the 160 days of growing season (from late April to early October, 2006), whereas the wild-type (WT) Kit flowered 55 days after germination (Figure 1*A* and 1*B*). We were able to produce seeds by moving this mutant plant to a controlled SDs. Plants from an F_2_ population derived from a cross of the mutant and WT segregated in field conditions into three categories based on their flowering time (days after germination): 56.9±1.8, 70.8±1.8 and never flowering mutants in a ratio of 1∶2∶1 (χ^2^[1∶2∶1] = 0.415<χ^2^_0.05,2_ = 5.99, *n* = 200). This result indicates that the mutation is semidominant and is controlled by a single gene. We named this locus *Ehd4* (*Early heading date 4*). Compared with WT, *ehd4* delayed flowering time by 49 d and 106 d under SDs and LDs, respectively (Figure 1*B*). Consistent with field observations, flowering time of the heterozygotes was also delayed under both SDs and LDs (Figure 1*B*). Notably, *ehd4* had a similar leaf emergence rate to WT under both SDs and LDs (Figure 1*C*), indicating that the late flowering phenotype is not caused by retardation in growth rate. The mature *ehd4* plants were taller, producing more but smaller seeds. The fertility of *ehd4* plants was similar to that of WT (Figure 1*D*–1*H*).

To test whether the delayed flowering phenotype is genetic background-dependent, we introduced the *ehd4* locus into Nip by backcrossing five times, followed by selfing. The *ehd4*-NIP plants (BC_5_F_3_) delayed flowering by 23 d under SDs compared to the WT NIP, but did not flower in NLDs or LDs (Figure 1*B*). Flowering time of the heterozygotes was also significantly delayed (Figure 1*B*). Thus, *ehd4* has a profound effect on flowering time, especially under LDs, in both a day-length neutral and a day-length sensitive genetic backgrounds.

### Molecular cloning of *Ehd4*

**Figure 2:**
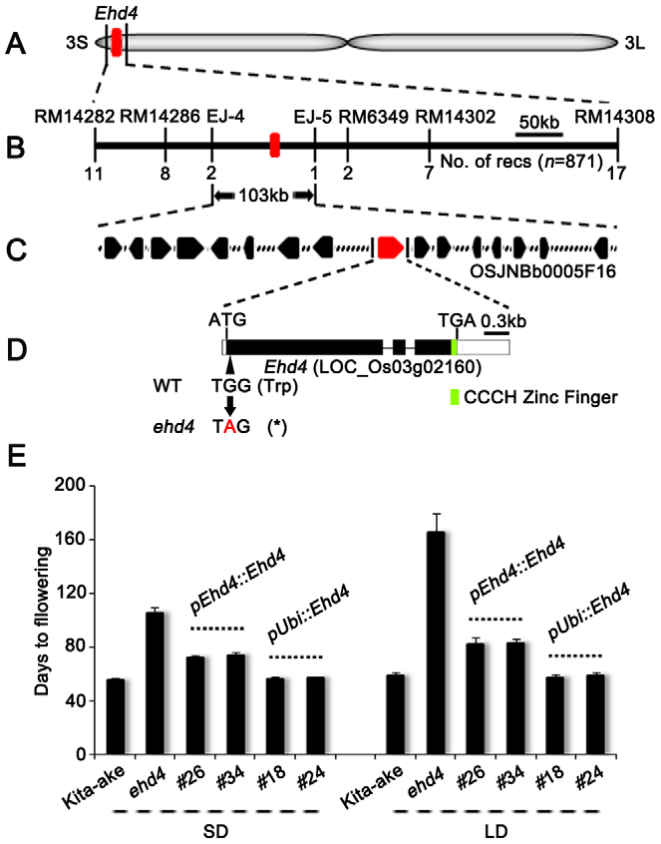
Map-based cloning of *Ehd4*. (*A*) Location of the *Ehd4* locus on rice chromosome 3. (*B*) High-resolution linkage map of *Ehd4*. (*C*) Candidate genes on BAC OSJNBb0005F16. (*D*) Structure of the *Ehd4* gene. Lines, black and white boxes represent introns, exons and untranslated regions, respectively. The base change from G to A creates an early stop codon (Asterisk). (*E*) Complementation of *ehd4*. *Ehd4* was driven by either the native promoter (*pEhd4::Ehd4*) or the maize *Ubiquitin-1* promoter (*pUbi::Ehd4*). T2 plants of two *pEhd4::Ehd4* lines (#26 and #34) and two *pUbi::Ehd4* lines (#18 and #24) were measured (*n* = 10). All plants were grown under both SD and LD conditions.

Flowering time control in rice is regulated by the interaction of multiple QTLs and the environments. Generally, flowering time of F2 population derived from *japonica*×*indica* displays a normal distribution pattern. Therefore, we crossed *ehd4* with 93-11, an *indica* variety with an available genome sequence [39], and generated a BC_1_F_2_ population for mapping the *ehd4* locus by backcrossing the F_1_ with 93-11. The *ehd4* locus was initially mapped to the short arm of chromosome 3 (Figure 2*A*). Using 871 extremely late flowering plants from approximately 25,000 BC_1_F_2_ plants grown in Hainan Island (18°48′N, 110°02′E, average day length 11 hours), South China, during the winter of 2008, we further delimited *Ehd4* to a 103 kb region, between the markers EJ-4 and EJ-5 (Figure 2*B*). This region contains 16 annotated ORFs (http://rapdb.dna.affrc.go.jp) (Figure 2*C*). Sequencing of the genomic DNA of all these genes revealed that there is a single nucleotide substitution (G to A) in the first exon of LOC_Os03g02160, which is predicted to encode a CCCH-Type zinc finger protein. The nucleotide change creates a premature stop codon at the very beginning of the predicted coding region (Figure 2*D*). Genomic sequence of this gene is identical between Kit and Nip (Figure S1). Quantitative real-time PCR (qRT-PCR) assay showed comparable expression of LOC_Os03g02160 in wild type, heterozygote and *ehd4* mutant plants (Figure S2). Transgenic plants carrying the full-length cDNA of LOC_Os03g02160, driven by the maize *Ubiquitin-1* promoter, fully complemented the *ehd4* phenotype under both SDs and LDs. Further, cDNA driven by its native promoter (2.7 kb upstream from ATG) also partially rescued the *ehd4* phenotype. The phenotypes of these transgenic lines (days to flowering) appeared to correlate with the expression level of *Ehd4* (Figure 2*E* and Figure S2). Thus, we concluded that the LOC_Os03g02160 locus corresponds to *Ehd4*.

### Expression of *Ehd4* is constitutive and diurnal

**Figure 3:**
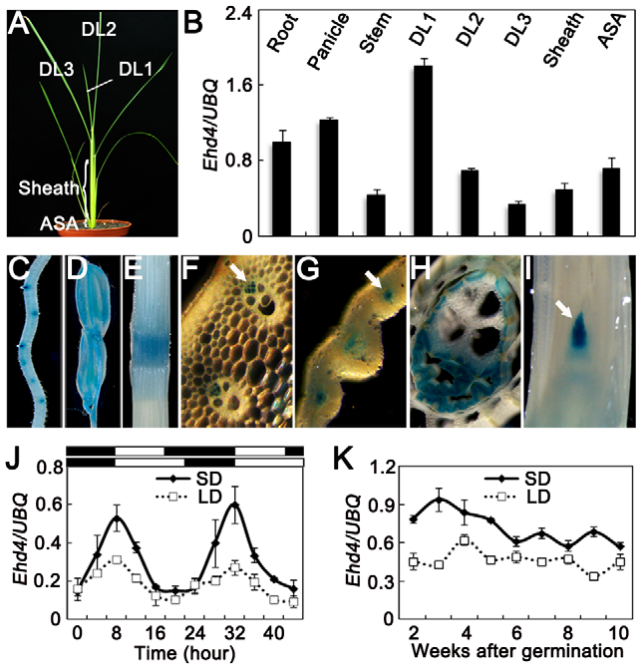
Expression pattern of *Ehd4*. (*A*) 30-d old wild-type plants (Kita-ake) grown under SDs were used for quantitative RT-PCR. DL1, newly emerging leaf; DL2, expending leaf; DL3, fully expended leaf; ASA, around the shoot apex. (*B*) *Ehd4* transcript levels in various organs (means±s.d, *n* = 3). (*C*) to (*I*) GUS staining of various organs in *pEHD4::GUS* transgenic plants. (*C*) Root; (*D*) Floret; (*E*) Stem; (*F*) to (*H*) Transverse sections of stem, immature leaf and sheath, respectively; (*I*) Longitudinal section of the shoot apical meristem (SAM). Arrow indicates phloem in (*F*) and (*G*) and SAM in (*I*). (*J*) and (*K*) Rhythmic and developmental expression of *Ehd4*. The rice *Ubiquitin-1* (*UBQ*) gene was used as the internal control. Values are shown as mean±s.d of three independent experiments and two biological replicates. The open and filled bars at the bottom represent the light and dark periods, respectively. s.d: standard deviations.

We examined the expression levels of *Ehd4* in various tissues and at different stages of leaf development (Figure 3*A*) by using qRT-PCR. *Ehd4* transcripts were detected in all tissues examined, but the highest expression was found in emerging young leaves and the lowest level in fully expanded leaves (Figure 3*B*). Histochemical staining of transgenic plants carrying the *GUS* reporter gene driven by the *Ehd4* promoter indicated that *GUS* was expressed in all tissues examined and was most abundant in the vascular tissue and apical meristem (Figure 3*C*–3*I*). The expression of *Ehd4* showed a diurnal expression pattern in leaves. It accumulates after dusk, reaching a peak at dawn, and damping rapidly thereafter under both SDs and LDs (Figure 3*J*). Moreover, *Ehd4* was expressed constantly during the vegetative growth from the second week to the 10th week after germination (Figure 3*K*).

### EHD4 may act as a transcriptional regulator

**Figure 4:**
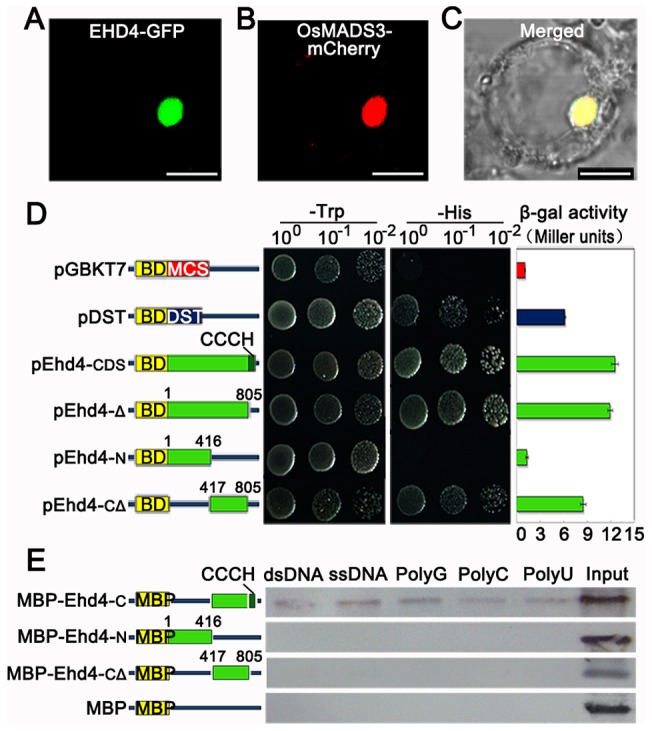
EHD4 is a nuclear protein with intrinsic transcriptional activation and nucleic acid binding activities. (*A*) Sub-cellular localization of EHD4-GFP fusion protein. (*B*) The nuclear marker MADS3-mCherry fusion protein. (*C*) Merged image of (*A*) and (*B*) under bright field. Scale bar = 10 µm in (*A*) to (*C*). (*D*) Transactivation assays of EHD4 and its deletion derivatives in the yeast GAL4 system. Full length EHD4 and several deletion derivatives of EHD4 (pEhd4-Δ, pEhd4-N and pEhd4-CΔ) were used in assays. The empty vector (BD-MCS) and BD-DST [56] were used as negative and positive control, respectively. Transformants were dropped onto SD/Trp- and SD/His- plates to allow growth of 48 hours before taking pictures. Values in β-galactosidase activity are means of three independent experiments. Bars stand for standard deviations. BD, DNA-binding domain of GAL4. (*E*) The CCCH motif is essential for binding to nucleic acids. C terminal, N terminal or C terminal without CCCH motif of EHD4 was expressed in *E*.*coli* and purified for binding assays. Deletion of the CCCH motif abolished the binding to ribohomopolymers and both double- and single-stranded calf thymus DNA.

In higher plants, CCCH-type zinc finger proteins have been shown to regulate gene expression by binding to DNA or RNA molecules in the nucleus [40]–[42]. We fused *Ehd4* with *GFP* and transiently expressed the EHD4-GFP fusion protein in rice leaf protoplasts. EHD4-GFP was exclusively co-localized with the OsMADS3-mCherry fusion protein (a nuclear marker) in the nucleus (Figure 4*A*–4*C*), indicating that EHD4 functions in the nucleus. We further fused EHD4 and its various deletions with the GAL4 DNA binding domain and investigated if EHD4 has transcriptional activation activity in yeast. Full-length wild type EHD4 and an EHD4 variant with only the CCCH motif removed were able to activate the reporter gene expression (Figure 4*D*). Further deletion of the C terminal region resulted in a dramatic reduction of the activation activity, whereas deletion of both the N-terminal and CCCH motif only had mild effects (Figure 4*D*). These observations suggest that the activation domain is located in the middle region close to the C-terminal of EHD4. In addition, a nucleic acid binding assay demonstrated that the C-terminal region, but not the N-terminal region, can bind to both double- and single-stranded calf thymus DNA and ribohomopolymers *in vitro*, and that removal of the CCCH motif from the C-terminal abolished the binding activity (Figure 4*E*). These results strongly support the notion that EHD4 likely functions as a transcriptional activator and that the CCCH motif is essential for its nucleic acid binding activity.

### *Ehd4* regulates expression of the “florigen” genes through *Ehd1*

**Figure 5:**
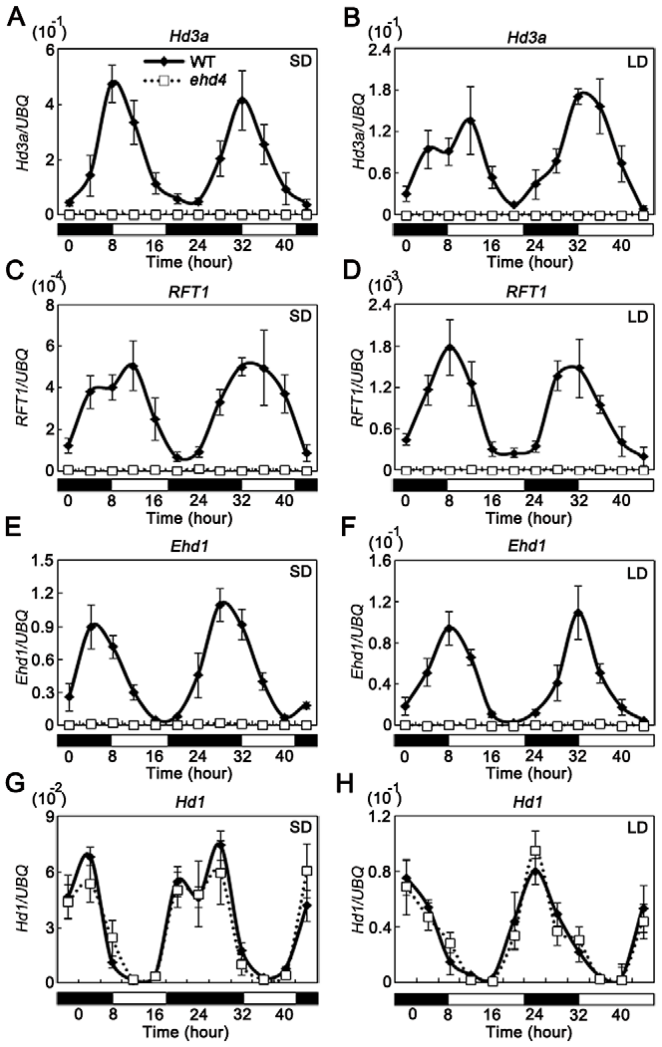
The rhythmic expression pattern of *Hd3a*, *RFT1*, and *Ehd1*, but not *Hd1*, was abolished in the *ehd4* mutant plants under both SDs and LDs. SDs (*A*, *C*, *E* and *G*); LDs (*B*, *D*, *F* and *H*). The open and filled bars at the bottom represent the light and dark periods, respectively. The rice *Ubiquitin-1* (*UBQ*) gene was used as the internal control. Values are shown as mean±s.d. (standard deviations) of three independent experiments and two biological replicates.

Photoperiodic induction of the floral transition in rice requires transcriptional activation of *Hd3a* and *RFT1*, the two ‘florigen’ genes, in leaves [20]–[22]. The diurnal expression pattern of *Ehd4* implies that it could be involved in photoperiodic control of flowering. To test this, we examined mRNA abundance of *Hd3a* and *RFT1* in *ehd4* and WT plants by qRT-PCR. The expression levels of *Hd3a* and *RFT1* were undetectable in *ehd4* under both SDs and LDs at all-time points examined during the 48 h period (Figure 5*A*–5*D*). Subsequently, expression of the downstream genes *OsMADS1*, *OsMADS14* and *OsMADS15* (three floral meristem identity genes; [22], [25]) was severely impaired in the *ehd4* mutants (Figure S3).

**Figure 6:**
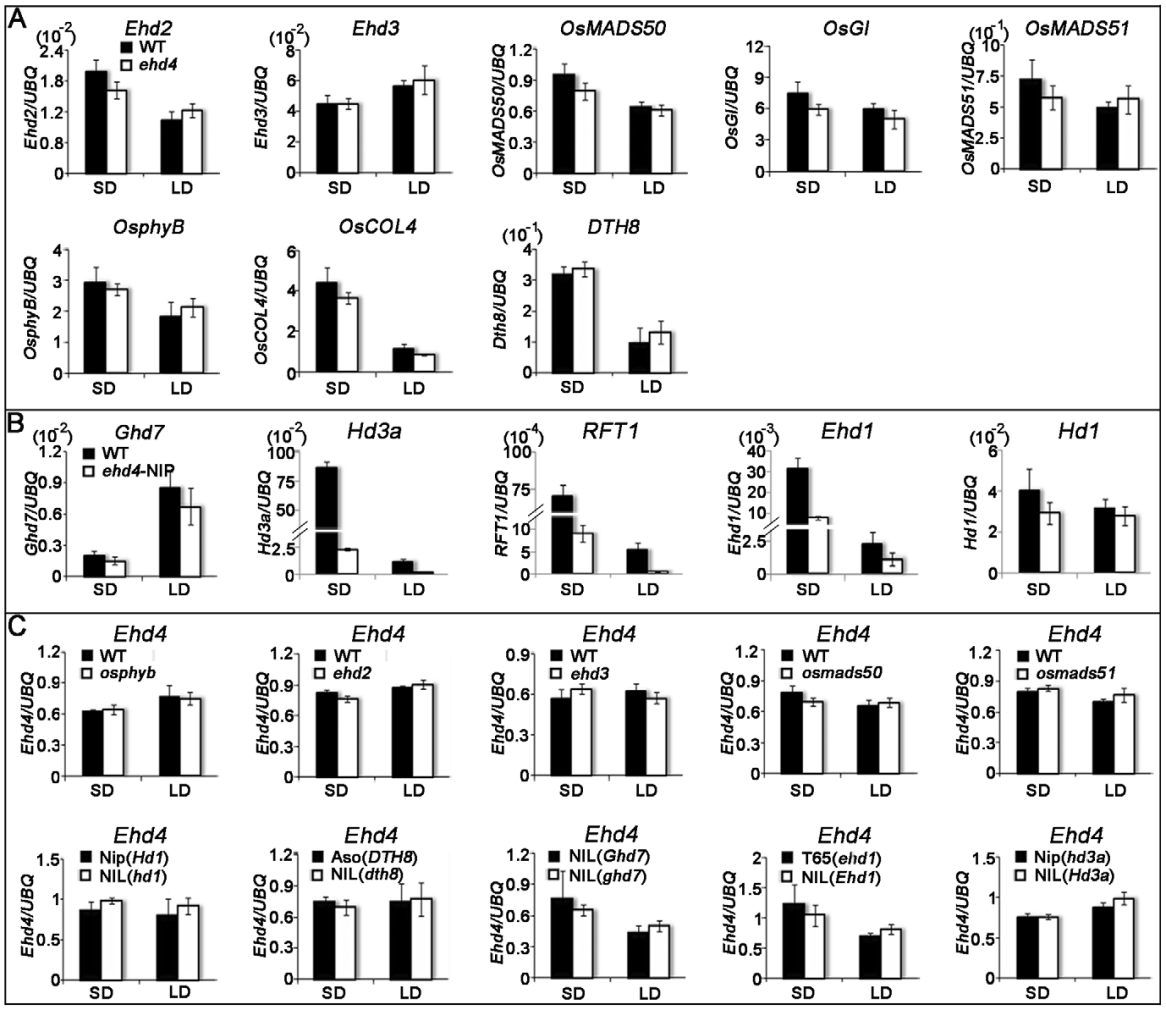
Quantitative RT–PCR analysis of representative flowering-related genes and *Ehd4* in various flowering-time mutants or their NILs (near-isogenic lines) and corresponding WTs under SDs and LDs. (*A*) Transcript level of *Ehd2*, *Ehd3*, *OsMADS50*, *OsGI*, *OsMADS51*, *OsphyB*, *OsCOL4* and *DTH8* in WT (Kita-ake) and *ehd4* plants. (*B*) Transcript level of *Ghd7*, *Hd3a*, *RFT1*, *Ehd1* and *Hd1* in WT (Nipponbare) and *ehd4*-*Nip* plants. (*C*) Transcript level of *Ehd4* in various flowering-time mutants or their NILs (near-isogenic lines) and corresponding WTs. Dongjin and the *osphyb* mutant [26]; Tohoku IL9 and the *ehd2* mutant [34]; Tohoku IL9 and the *ehd3* mutant [36]; Dongjin and the *osmads50* mutant [31]; Dongjin and the *osmads51* mutant [30]; Nipponbare and a NIL carrying a non-functional *Hd1* allele [19]; Asominori and a NIL carrying a nonfunctional *DTH8* allele [29]; A NIL carrying a functional *Ghd7* allele and a NIL carrying a non-functional in the Shanyou 63 background [28]. Taichun 65 carrying a non-functional *Ehd1* allele and a NIL carrying a functional *Ehd1* allele [24]; Nipponbare carrying a partially functional *Hd3a* allele and a NIL carrying a functional *Hd3a* allele [20]; Penultimate leaves were harvested around reported peak expression level of each gene during the 24 hrs photoperiod - at dawn for *OsphyB*, *OsCOL4*, *Ehd1*, *Ehd2*, *Hd3a*, *RFT1* and *Ehd4*, 3 h after dawn for *Ghd7*, 8 h after dawn for *Ehd3*, *OsMADS50*, *OsMADS51* and *DTH8* and immediately after dusk for *OsGI* and *Hd1* from 28 d-old (SDs) and 35 d-old (LDs) plants. The rice *Ubiquitin-1* (*UBQ*) gene was used as the internal control. Values are shown as mean±s.d (standard deviations) of three independent experiments and two biological replicates.

*Hd1* and *Ehd1* are known to regulate *Hd3a* and *RFT1* [19], [24]. To investigate whether the activation of ‘florigen’ genes by *Ehd4* is mediated by *Hd1* and/or *Ehd1*, we compared their mRNA levels between *ehd4* and WT plants. Strikingly, expression of *Ehd1*, but not *Hd1*, was abolished in *ehd4* mutants, indicating that *Ehd4* is essential for *Ehd1* expression (Figure 5*E*–5*H*). *Ehd4* has a diurnal expression pattern similar to that of *Ehd1*, typically peaking at dawn (Compare Figure 3*J* with Figure 5*E* and 5*F*). Next, we examined whether *Ehd4* affects the expression of other known regulators of *Ehd1*. To our surprise, the transcription levels of five positive regulators (*Ehd2*, *Ehd3*, *OsMADS50*, *OsGI* and *OsMADS51*) and four negative regulators of *Ehd1* (*OsphyB*, *OsCOL4*, *DTH8* and *Ghd7*) were not significantly affected in *ehd4* (Figure 6*A* and 6*B*). These observations suggest that *Ehd4* functions upstream of *Ehd1*, but largely independent of other known regulators of *Ehd1.* Consistent with this, down regulation of *Ehd1*, *Hd3a* and *RFT1* in *ehd4* was also seen in the Nipponbare background and constantly seen at different stages during plant development (Figure 6*B* and Figure S4).

**Figure 7:**
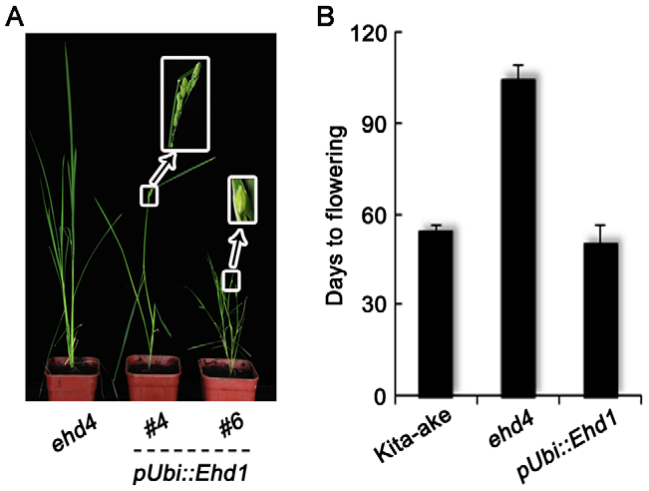
Complementation of *ehd4* by over-expression of *Ehd1*. (*A*) Phenotypes of the mutant and transgenic plants at heading stage. (*B*) *Ehd1*, when driven by the maize *Ubiquitin-1* promoter (*pUbi::Ehd1*), was able to rescue the flowering phenotype of *ehd4* (T0 plants, *n* = 8). All plants were grown under SDs.

To investigate whether *Ehd4* expression is regulated by other flowering genes, we examined the expression of *Ehd4* in *osphyb*, *ehd2*, *ehd3*, *osmads50* and *osmads51* mutants and near-isogenic lines (NILs) which carrying a deficient *Hd1*, *Ghd7* or *DTH8* alleles. Notably, we detected no significant differences of *Ehd4* expression in these mutants or NILs, as compared to their corresponding WT plants (Figure 6*C*). In addition, no significant change of *Ehd4* expression was seen in NILs deficient in *Ehd1* or *Hd3a* either (Figure 6*C*). These results suggest that *Ehd4* acts independent of other *Ehd1* regulators we examined. Together, these observations suggest that *Ehd4* regulates the expression of *Hd3a* and *RFT1* through *Ehd1*. This notion was also supported by the observation that over-expression of *Ehd1* fully rescued the late flowering phenotype of *ehd4* under SDs (Figure 7).

Since EHD4 has a transcriptional activation and nucleic acid binding activity and it promotes *Ehd1* expression, we next carried out a yeast one-hybrid assay and a transient transcription assay [43], [44] to test whether *Ehd1* is a direct downstream target of EHD4. However, only OsLFL1 [45], but not EHD4, was able to interact with the *Ehd1* promoter (Figure S5), indicating that *Ehd1* is likely an indirect target of *Ehd4.* In addition, yeast three-hybrid assay [46] also failed to detect a direct binding of EHD4 to the *Ehd1* mRNA (Figure S6). Moreover, neither EHD2 nor EHD3, directly binds to the *Ehd1* promoter (Figure S5). Our yeast two-hybrid assay showed that there was no direct interaction among the EHD2, EHD3 and EHD4 proteins (Figure S7). Together, these results suggest that *Ehd2*, *Ehd3* and *Ehd4* likely act through distinct pathways to promote the expression of *Ehd1*.

### Transcriptome analysis of *ehd4* plants

To further reveal the molecular basis of the flowering phenotype of *ehd4*, we performed a transcriptome analysis of *ehd4* and wild-type plants using RNA-seq to identify genes downstream of *Ehd4*. RNA samples were extracted from the penultimate leaves (collected at dawn) of 30 d-old *ehd4* and WT plants (Kita-ake) grown under LDs. We obtained 2.5 M tags and found a total of 256 genes altered in expression with an estimated false-discovery rate of 0.1% and the absolute value of log_2_Ratio at 3.15 under the Bayesian model (Table S1; [47]). We found that the transcript numbers of *Hd3a*, *RFT1*, *Ehd1*, *OsMADS1*, *OsMADS14* and *OsMADS15* reduced dramatically in *ehd4* plants (Table S1), consistent with the qRT-PCR results (Figure 5*A*–5*F* and Figure S3). Our qRT-PCR analysis with other four genes (with a log_2_ Ratio of −11.73, −10.33, −5.80 and −3.15, respectively) also further confirmed the reliability of the RNA-seq results (Figure S8). Notably, we found that among the genes down-regulated in *end4,* 25 of them are known or putative transcription factors, including MADS box, Zinc finger, MYB, SBP and B3 proteins (Table S1). These genes could be potential candidates involved in the *Ehd4*-*Ehd1*-*Hd3a/RFT1* pathway to regulate photoperiodic flowering in rice.

### *Ehd4* is unique and highly conserved in rice

*Ehd4* is a single copy gene in the rice genome. It is predicted to code for a polypeptide of 832 amino acids long, which contains a CCCH (C-X_7_-C-X_5_-C-X_3_-H)-type zinc finger motif at the C-terminus (Figure S9). A blast search (http://www.ncbi.nlm.nih.gov/) found that EHD4 has no clear homologs in other plant or animal species. Thus it appears that *Ehd4* represents a unique regulator of flowering time in rice.

**Figure 8:**
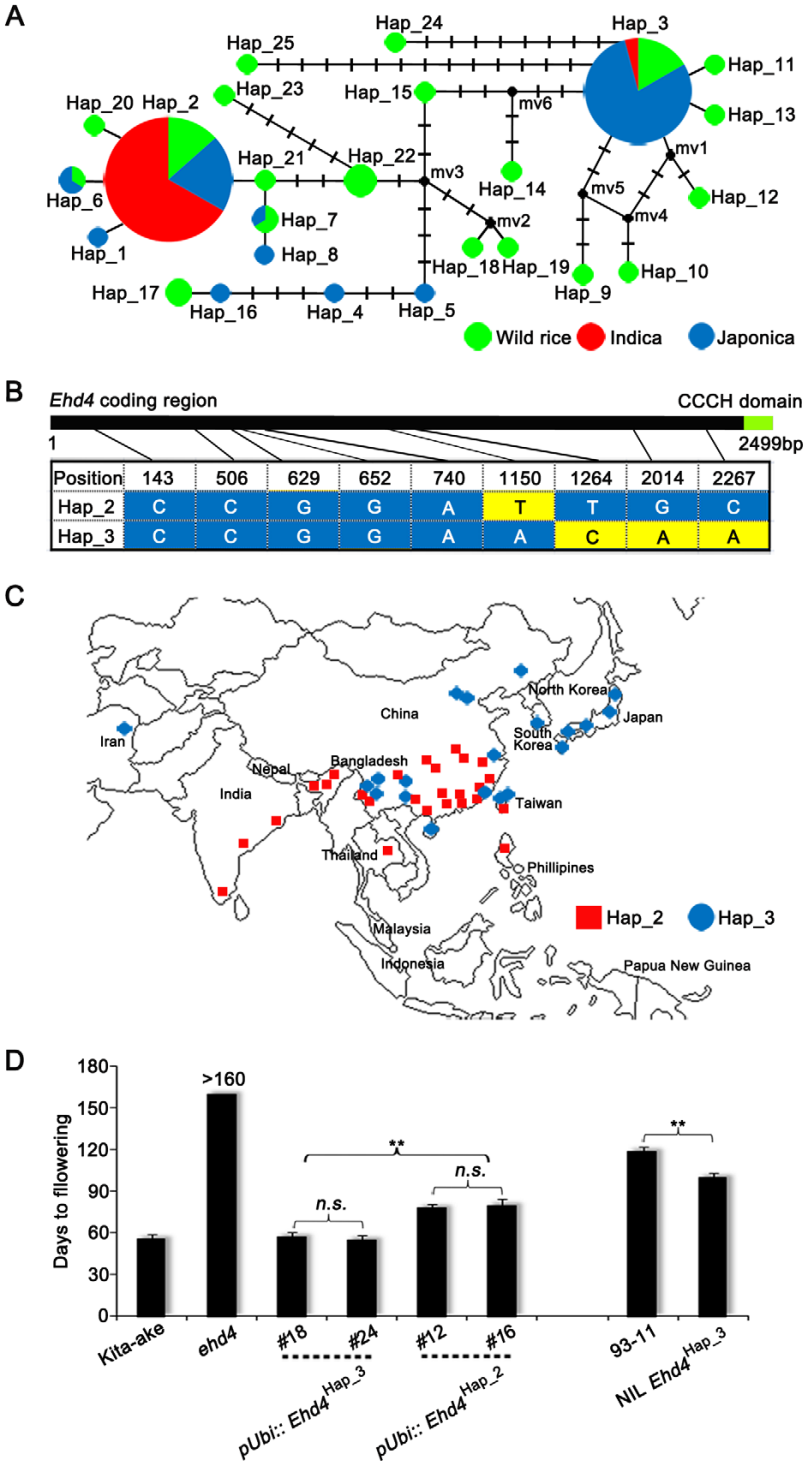
Natural variations in the *Ehd4* coding region among rice germplasm core collection. (*A*) Haplotype network of the *Ehd4* alleles in 86 rice accessions. Haplotype frequencies are proportional to the area of the circles. The proportion of wild rice and two cultivated subgroups (*indica* and *japonica*) in each haplotype is represented by different colors. (*B*) The polymorphic nucleotides of Hap_2 and Hap_3 of *Ehd4* gene in the core collection. The number on the top shows the position of nucleotide polymorphisms in the coding region starting from the ATG start codon. (*C*) Geographic distribution of the cultivated rice accessions belonging to Hap_2 and Hap_3. (*D*) Flowering time of transgenic plants carrying two major haplotypes of *Ehd4* driven by the maize *Ubiquitin-1* promoter in *ehd4* (Kita-ake background) and NIL carrying *Ehd4* ^Hap3^ compared with the 93-11 parental plants. T2 plants of two *pUbi::Ehd4* ^Hap3^ (#18 and #24) and two *pUbi::Ehd4* ^Hap2^ (#12 and #16) lines were measured (*n* = 15). All plants were grown in the natural long day field conditions. Values are means±s.d. (standard deviations) (*n* = 15). **Significant at 1% level; *n.s.*, not significant.

To investigate the evolutionary history of the gene in rice, we analyzed the *Ehd4* sequences from 86 rice accessions with wide geographic distribution and diverse genetic backgrounds, including 32 wild rice species (*O*. *rufipogon* and *O*. *nivara*) and 54 cultivated rice (Table S2; [48]). *Ehd4* appears to be highly conserved across these accessions (share >99.2% or higher amino acid sequence identities) (Table S2). Sequence analysis identified 25 haplotypes among these accessions. Strikingly, 21 haplotypes were identified in 32 wild rice accessions (*O*. *rufipogon* and *O*. *nivara*) but only 8 haplotypes were identified in 54 cultivated rice accessions analyzed in this study. The dramatic reduction in genetic diversity at this locus suggests that *Ehd4* might subject to bottleneck effect [49]. Notably, 4 haplotypes (Hap_2, 3, 6 and 7) are shared in cultivated and wild rice (Figure 8*A*, Table S2), and among them, Hap_2 and Hap_3 together account 25% of the 32 wild rice and 85% of the 54 cultivated rice (*O. sativa*) respectively (Figure 8*A*, 8*B* and Table S2). Among the cultivated rice, 20 (77%) *indica* and 6 (23%) *japonica* accessions belong to Hap_2, while 19 (95%) *japonica* and 1 (5%) *indica* accessions belong to Hap_3 (Figure 8*A* and Table S2). This result suggests that the Hap_2 and Hap_3 represent the two major haplotypes at the *Ehd4* locus in cultivated rice and that they exist in wild rice before domestication. The distribution pattern of Hap_2 and Hap_3 in *indica* (mostly distributed in lower latitude and elevation zones) and *japonica* (mostly distributed in higher latitude and elevation zones) (Figure 8*C*) implies a likely correlation between the geographic distribution and the functional differences of *Ehd4* haplotypes among these cultivated accessions analyzed.

To test the possible functional differences of these two haplotypes, we introduced the full-length *Ehd4* cDNA of *indica* variety 93-11 (Hap_2) and Kita-ake (a *japonica* landrace, Hap_3) driven by the maize *Ubiquitin-1* promoter into the *ehd4* mutant (Kita-ake background). Strikingly, over-expression of Hap_3, but not Hap_2, fully complemented the *ehd4* phenotype under NLDs, although their expression levels are comparable (Figure 8*D* and Figure S10). This result suggests that Hap_3 of *Ehd4* is functionally more potent in promoting flowering than Hap_2. As another test of this notion, we introduced the Hap_3 allele from Kita-ake into 93-11 by backcrossing five times, followed by selfing. Strikingly, the NIL *Ehd4* ^Hap_3^ plants (BC_5_F_3_) flowered earlier by 19 d under NLDs compared to the parental 93-11 plants (Figure 8*D*). Together, these results suggest that the functional differences of *Ehd4* haplotypes might play a role in geographic adaptation of cultivated rice.

## Discussion

In this study, we have uncovered *Ehd4*, which codes for a CCCH-type zinc finger protein essential for promoting flowering under both SD and LD conditions in rice, irrespective of genetic backgrounds. We demonstrated that *Ehd4* promotes flowering by positively regulating the expression of *Hd3a* and *RFT1* through *Ehd1* but independent of other known important *Ehd1* regulators. We further showed that the late-flowering phenotype of *ehd4* is more profound in Kita-ake (Kit) (a day-length neutral variety) than in Nipponbare (Nip) (a day-length sensitive variety) under SDs, but *ehd4* plants flowered eventually in Kit (164 days after germination) but not in Nip under LDs (Figure 1*B*). It is known that *Hd1* acts to promote flowering under SDs but delay flowering under LDs [19]. We found that *Hd1* has a 36-bp insertion and two SNPs in Kit compared to Nip (Figure S1), implying that in Kit, the promoting role under SDs and repressing role under LDs of *Hd1* may be impaired. This may at least partially explains why the late-flowering phenotype of *ehd4*-Nip is less severe under SDs but more severe under LDs. In addition, we found that Kit carries a truncated allele of *Ghd7*, a repressor of *Ehd1* under LDs, whereas Nip carries a partially functional *Ghd7* allele (Figure S1; [28]). Therefore, the never-flowering phenotype of *ehd4* in Nip under LDs could be due to the repressive effect of *Hd1* and *Ghd7*. Strikingly, even in the absence of the functional *Hd1* and *Ghd7* alleles in Kit, *ehd4* alone delayed flowering time by three folds under LDs (Figure 1*B*), suggesting that *Ehd4* plays a major role in promoting flowering in rice, particularly under LDs.

Previous studies revealed that rice *Ehd1* is a critical convergence point of flowering time regulation by multiple signaling pathways and that *Ehd1* acts independently of *Hd1*. *Ehd1* encodes a B-type response regulator that is highly conserved in cultivated rice, but has no homolog in Arabidopsis [23], [24]. Up to date, 12 genes have been shown to regulate *Ehd1* expression, including 5 positive regulators (*Ehd2*, *Ehd3*, *OsGI*, *OsMADS50* and *OsMADS51*) and 7 negative regulators (*SE5*, *OsphyB*, *Ghd7*, *DTH8*, *OsLFL1*, *OsCOL4* and *OsMADS56*). We demonstrated that *Ehd4* promotes flowering by positively regulating the expression of *Hd3a* and *RFT1* through *Ehd1*, but independently of these known *Ehd1* regulators.

It is also of interest to note that the majority of *Ehd1* regulators uncovered thus far are nuclear proteins and many of them act as transcriptional regulators, including GHD7, DTH8, OsMAD50, OsMAD51, EHD2, EHD3, OsLFL1, OsMAD56 and OsCOL4 [26], [28]–[34], [36], [45], [50]. Map-based cloning revealed that *Ehd4* encodes a novel CCCH-type zinc finger protein also localized to the nucleus. The CCCH-type zinc finger protein family is defined as a group of proteins containing 1–6 copies of the canonical C-X-C-X-C-X-H motif (C-X_6–14_-C-X_4–5_-C-X_3_-H, where X is any amino acid) [51]. This type of proteins has been found in organisms ranging from human to yeast and many of them have been shown to have either an RNA binding function involved in RNA processing or DNA binding activity [40]–[42]. There are at least 68 CCCH-type genes in Arabidopsis and 67 in rice, respectively [52]. However, only a few plant CCCH proteins have been functionally characterized. EHD4 is the first CCCH-type protein found to regulate photoperiodic flowering. We found that EHD4 is capable of binding to nucleic acids *in vitro* and transactivate transcription in yeast, suggesting that it likely functions as a transcription factor. Further, our transcriptome analysis revealed that a significant portion of *Ehd4*-regulated downstream genes are also transcription factors, including several previously identified flowering regulators, such as *Ehd1*, *OsLFL1 OsMADS1*, *OsMADS14* and *OsMADS15*, and other putative transcription factors, including MADS box, Zinc finger, MYB, SBP and B3 proteins (Table S1). These findings together suggest that transcriptional regulation plays a critical role in photoperiodic regulation of flowering in rice. However, despite we have demonstrated that EHD4 has double-stranded DNA and ribohomopolymer binding activity and transactivation activity in yeast, we have not been able to identify the direct target genes of EHD4 in this study. It is also possible that EHD4 may bind to RNA molecules and degrades transcripts of unknown *Ehd1* repressors. Further studies are required to elucidate the biochemical function of EHD4 and its functional relationship with other nuclear regulators of flowering time.

Rice is known as a short day plant. However, cultivated rice (*O. sativa*) is grown widely in Asia, with a northern limit of nearly 53°N in northern Asia (Northern provinces of China and Korea, where natural day length during rice cultivation is nearly 15 hours light; [53]), whereas *O. rufipogon*, a wild rice that is the most relative ancestor of *O. sativa*, is mainly distributed at tropical latitudes with a northern limit about 28°N [54]. The northward expansion of cultivated rice into higher latitudes must be accompanied by human selection of the flowering time trait during rice domestication and breeding, to secure a harvest before cold weather approaches. Strikingly, *Ehd4* has no obvious homologs in other plant species including Arabidopsis, maize and sorghum, suggesting that *Ehd4* originated along with the diversification of the *Oryza* genus from the grass family during evolution. Amino acid sequence comparison of EHD4 showed identities at 99.2% or higher among a core collection of rice germplasm with wide geographic distribution and diverse genetic backgrounds, including wild rice species ([48]; Table S2). Interestingly, we found two major haplotypes of *Ehd4*, Hap_2 (the major haplotype in *indica*) and Hap_3 (the major haplotype in *japonica*) and that Hap_3 is functional more potent in promoting flowering under NLDs. Since *indica* rice is known to distribute mostly in lower latitude and elevation zones (between the latitude 3°S-35°N), while *japonica* varieties are mostly distributed in higher latitude and elevation zones (between the latitude 15°N-53°N), our findings suggest that *Ehd4* may have contributed to the northward expansion and regional adaptation of cultivated rice into higher latitudes.

## Materials and Methods

### Plant material and growth conditions

The *ehd4* mutant was initially identified from a tissue culture-derived population of rice cv Kita-ake (*japonica*) under natural-day conditions in a paddy field in Beijing (39°54′N, 116°23′E), China (2006). To generate *ehd4-nip* plants, the mutant locus was introgressed into Nipponbare (*japonica*) background by crossing and backcrossing for five generations (BC_5_), where *ehd4* mutant is the donor parent and Nipponbare is the recurrent parent, by using marker assisted selection (MAS).

Plants were grown in controlled-growth chambers (Conviron) under SDs (10 h light at 30°C/14 h dark at 25°C) or LDs (14.5 h light at 30°C/9.5 h dark at 25°C) with a relative humidity of ∼70%. The light intensity was ∼800 µmol m^−2^ s^−1^.

### Map-based cloning

To map the *ehd4* locus, the mutant was crossed with the *indica* cv 93-11 and then the F_1_ plants were backcrossed with 93-11 to produce a BC_1_F_2_ population. We used two DNA pools generated from 15 BC_1_F_2_ late-flowering and 15 normal plants, respectively, for rough mapping. For fine mapping, 871 never-flowering plants segregated in the BC_1_F_2_ population were used.

### Vector construction and plant transformation

For the complementary test, the *Ehd4* full-length cDNA driven by its native (2.7-kb) or the maize *Ubiquitin-1* (*Ubi*) promoter were cloned into the binary vector pCAMBIA1390 by using In-Fusion Advantage PCR Cloning Kits (Clontech) to create *pEhd4::Ehd4* and *pUbi::Ehd4*, respectively. The 2.7-kb long promoter was also cloned into pCAMBIA-1305.1 to create *pEhd4::GUS*. The resultant plasmids were transformed into the *Agrobacterium tumefaciens* strain *EHA105* and then introduced into *ehd4* (for complementary test) or Kita-ake WT plants (*pEhd4::GUS*). At least 15 transgenic events were produced for each construct.

### Subcellular localization of EHD4

GFP was fused to the C-terminus of EHD4 under the control of the 35S CaMV promoter in the pA7 vector. The EHD4-GFP fusion and the nucleus marker OsMADS3-mCherry were transiently co-expressed in rice leaf protoplasts by PEG (polyethylene glycol) treatment [55]. Fluorescence was observed using a Leica TCS-SP4 confocal microscope.

### Transactivation activity assay

Transactivation activity assay was performed using the Matchmaker GAL4 Two-Hybrid System 3 (Clontech). Plasmids containing GAL4 DNA binding domain fused with EHD4 deletions were transformed into the yeast strain AH109. The substrate chlorophenol red-β-D-galactopyranoside (CPRG; Roche Biochemicals) was used to measure the β-galactosidase activity according to the Yeast Protocols Handbook (Clontech).

### *In vitro* nucleic acid binding assay

EHD4 deletions were cloned into the pMAL-c2x (NEB) and expressed in *E. coli.* 0.5 mg purified protein was incubated with 20 mL of poly rG, poly rC, or poly rU attached to agarose beads or double- or single-stranded calf thymus DNA attached to cellulose beads (Sigma) in 500 mL of RHPA binding buffer (10 mM Tris, pH 7.4, 2.5 mM MgCl_2_, 0.5% Triton X-100, NaCl at various concentrations) with 1 mg/mL heparin. After incubation at 4°C for 10 min, the beads were washed five times in the RHPA buffer and then boiled in the SDS loading buffer. Binding of fusion proteins to RNA or DNA was confirmed by protein gel blot using anti-MBP antibodies (NEB).

### Yeast one-hybrid assay

Yeast one-hybrid assay was performed according to the method described in [43]. To generate GAD-EHD4, GAD-EHD2, GAD-EHD3 and GAD-OsLFL1, their full-length cDNAs were cloned into pJG4-5 vector. To generate the *Ehd1p::LacZ* reporter gene, a 3.2 kb fragment of *Ehd1* promoter (including the 5′-UTR) was amplified from Nipponbare genomic DNA and inserted into the corresponding sites of the reporter plasmid pLacZi2μ. Plasmids were co-transformed into the yeast strain EGY48. Transformants were grown onto SD/Trp-/Ura plates for 48 hours and then transferred onto X-gal (5-bromo-4-chloro-3-indolyl-β-D-galactopyranoside) plates for blue color development.

### Yeast three-hybrid assay

Yeast three-hybrid assay was performed according to the method described in [46]. Full-length *Ehd4* cDNA was cloned into pACTII vector to generate GAD-EHD4. A series DNA fragments (about 140 bp) that transcripting *Ehd1* mRNA sequence were cloned into pMS2-1 vector. Plasmids were co-transformed into the yeast strain YBZ-1. Transformants were grown on plates containing selective media (SD/Ura-/Leu-/His-+0, 2 or 8 mM 3-aminotriazole) for 48 hours before assay.

### Bioluminescence assays

Isolation of rice leaf protoplast and PEG-mediated transfection were performed as described [55]. The reporter construct pGreen-*Ehd1*p-LUC and effector plasmids (pEGAD-MycEHD4, EHD2, EHD3 or OsLFL1) were co-transformed into protoplasts. After transformation, the protoplasts were incubated in darkness for 12–16 h. Bioluminescence assay was performed according to the method described in [44].

### Quantitative real-time RT-PCR

Total RNA was extracted using the RNeasy Plant Mini Kit (QIAGEN). For quantitative real-time RT-PCR, the first-strand cDNA was synthesized using the QuantiTect Reverse Transcription Kit (QIAGEN) and then PCR was performed using gene-specific primers and SYBR Premix ExTaq reagent (Takara) with an ABI Prism 7900 HT Sequence Detection System (Applied Biosystems) according to the manufacturer's instructions. PCR reactions were carried out in triplicate for each sample from two independent biological replicates and the rice *Ubiquitin-1* gene was used as the internal control.

### RNA–seq (next-generation sequencing of RNA)

We used the Illumina HiSeq 2000 Genome Analyzer to get tags with CATG site, in which the adapter sequences are 2*100 bp. With Illumina's digital gene expression assay, we obtained 11.7 million sequence tags per sample. After removing low quality reads and low quality bases of quality value, clean reads were mapped to the *O. ssp*. *japonica* reference sequences using SOAPaligner/soap2. Mismatches of not more than one base were allowed in the alignment and we generated 8.9 million perfect match tags (76.08%) for each sample. Initially, we determine 27020 genes of significant differences in expression between the groups of wild-type and mutants by a Student's *t*-test. With a dedicated Bayesian model, we found 256 transcripts of differential expression with an estimated false-discovery rate of 0.1% and the absolute value of log^2^Ratio is more than 3.15.

All primers used in this study are listed in Table S3.

### Accession numbers

Data deposition: The *Ehd4* sequence reported in this paper has been deposited in the GenBank database accession no. JQ828863 (cDNA).

## Supporting Information

Figure S1 Alignment of the open reading frames of eleven flowering related genes between Kita-ake and Nipponbare. Kasa, Kasalash. SM, synonymous mutations; Asterisks represent the premature stop codon.
(JPG)

Figure S2 Transcript levels of *Ehd4* in WT (Kita-ake), heterozygote (HETE), *ehd4* and transgenic plants. Penultimate leaves were harvested at dawn from 28 d-old (SDs) and 35 d-old (LDs) plants. The rice *Ubiquitin-1* (*UBQ*) gene was used as the internal control. Values are shown as mean±s.d (standard deviations) of three independent experiments and two biological replicates.
(JPG)

Figure S3 Transcript levels of *OsMADS14*, *OsMADS15* and *OsMADS1* in WT (Kita-ake) and *ehd4* plants. Penultimate leaves were harvested around reported peak expression level of each gene during the 24 hrs photoperiod - at dawn from 28 d-old (SDs) and 35 d-old (LDs) plants. The rice *Ubiquitin-1* (*UBQ*) gene was used as the internal control. Values are shown as mean±s.d (standard deviations) of three independent experiments and two biological replicates.
(JPG)

Figure S4 Developmental expression pattern of *Ehd1*, *Hd3a* and *RFT1* in WT (Kita-ake) and *ehd4* plants under SDs (*A*, *C* and *E*) and LDs (*B*, *D* and *F*). The rice *Ubiquitin-1* (*UBQ*) gene was used as the internal control in the quantitative RT-PCR analysis. Values are shown as mean±s.d. (standard deviations) of three independent experiments and two biological replicates.
(JPG)

Figure S5 Yeast One-Hybrid and Bioluminescence assays. (*A*) GAD-LFL1 (positive control; [45]), but not GAD-EHD2, GAD-EHD3, GAD-EHD4 or GAD itself (negative control), strongly activate expression of the *LacZ* reporter genes driven by the *Ehd1* promoter (3.2 kb upstream of the ATG start codon) in yeast one-hybrid assay. (*B*) Structure of the vector used for transient expression. 35S, 35S CaMV promoter; REN, renilla luciferase; 35S mini, 35S CaMV minimum promoter; LUC, luciferase gene; 35S term, 35S CaMV terminator. (*C*) Relative reporter activity (LUC/REN) in rice protoplasts. Bioluminescence assays showing that expression of *Ehd1::LUC* reporter was not induced by EHD4, EHD2, EHD3 or GAD (empty vector) itself but strongly repressed by LFL1 (positive control; [45]) in rice leaf protoplasts. The relative LUC activities normalized to the REN activity are shown (LUC/REN, *n* = 3).
(JPG)

Figure S6 Yeast Three-Hybrid assay. (*A*) Genomic structure of the *Ehd1* locus. Regions used for assays in (*B*) are underlined and numbered in order. (*B*) Yeast Three-Hybrid assays showing that EHD4 did not interact with any part of *Ehd1* mRNA. The plasmid pIIIA/IRE-MS2 expressing 5′ IRE-MS2 3′ hybrid RNA from the yeast RNAseP promoter and the plasmid pAD-IRP expressing the rabbit Iron Regulatory Protein fused to the Gal 4 Activation Domain was used as the positive control [46] and the corresponding empty vectors were used as the negative control.
(JPG)

Figure S7 Yeast Two-Hybrid Assay. Yeast two-hybrid assays showing that EHD2, EHD3 and EHD4 did not interact with each other. BD-OsMADS50 and AD-OsMADS56 were used as the positive control [50] and the empty vector were used as the negative control. ND, not determined.
(JPG)

Figure S8 qRT-PCR confirmation of RNA-seq results. Four genes with reduced expression in *ehd4* as determined by RNA-seq, were chosen for qRT-PCR assay. Independent penultimate leaves of 28 d-old plants grown under LDs were collected at dawn. The rice *Ubiquitin-1* (*UBQ*) gene was used as the internal control. Values are shown as mean±s.d (standard deviations) of three independent experiments.
(JPG)

Figure S9 Alignment of the CCCH motif of EHD4 with the zinc fingers from other CCCH-type proteins. Zinc fingers are from rice OsLIC (Os06g49080) and OsDOS (Os01g09620), Arabidopsis FES1 (At2g33835), HUA1 (NP_187874), PEI (S22126), SOMNUS (At1g03790), SZF1 (At3g55980) and SZF2 (At2g40140), Cotton GhZFP1 (AY887895), C. elegans MEX-1 (U81043), PIE-1 (AAB17868) and POS-1 (T37246), human TTP (P26651) and yeast ZFS1(P47979). CCCH motifs from the same gene are shown as serial numbers. The consensus CCCH residues are shaded with yellow color. Other identical or similar residues are shaded with blue or purple color, respectively.
(JPG)

Figure S10 Transcript levels of *Ehd4* in WT (Kita-ake), *ehd4* and transgenic plants. Penultimate leaves were harvested at dawn from 35 d-old plants grown under natural long day conditions in Beijing. The rice *Ubiquitin-1* (*UBQ*) gene was used as the internal control. Values are shown as mean±s.d (standard deviations) of three independent experiments and two biological replicates.
(JPG)

Table S1 Transcriptome analysis of *ehd4* plants. Genes with expression level changes of at least 8 fold (*ehd4*/Wild Type) were listed.
(DOC)

Table S2 Summary of the *Ehd4* allele types in cultivated and wild rice.
(DOC)

Table S3 Primers used in this study.
(DOC)
