# Supplementary figures and images for: Ehd4 Encodes a Novel and Oryza-Genus-Specific Regulator of Photoperiodic Flowering in Rice
Source: PLoS Genet. 2013 Feb 21;9(2):e1003281. doi: 10.1371/journal.pgen.1003281 (PMC3578780; doi:10.1371/journal.pgen.1003281)

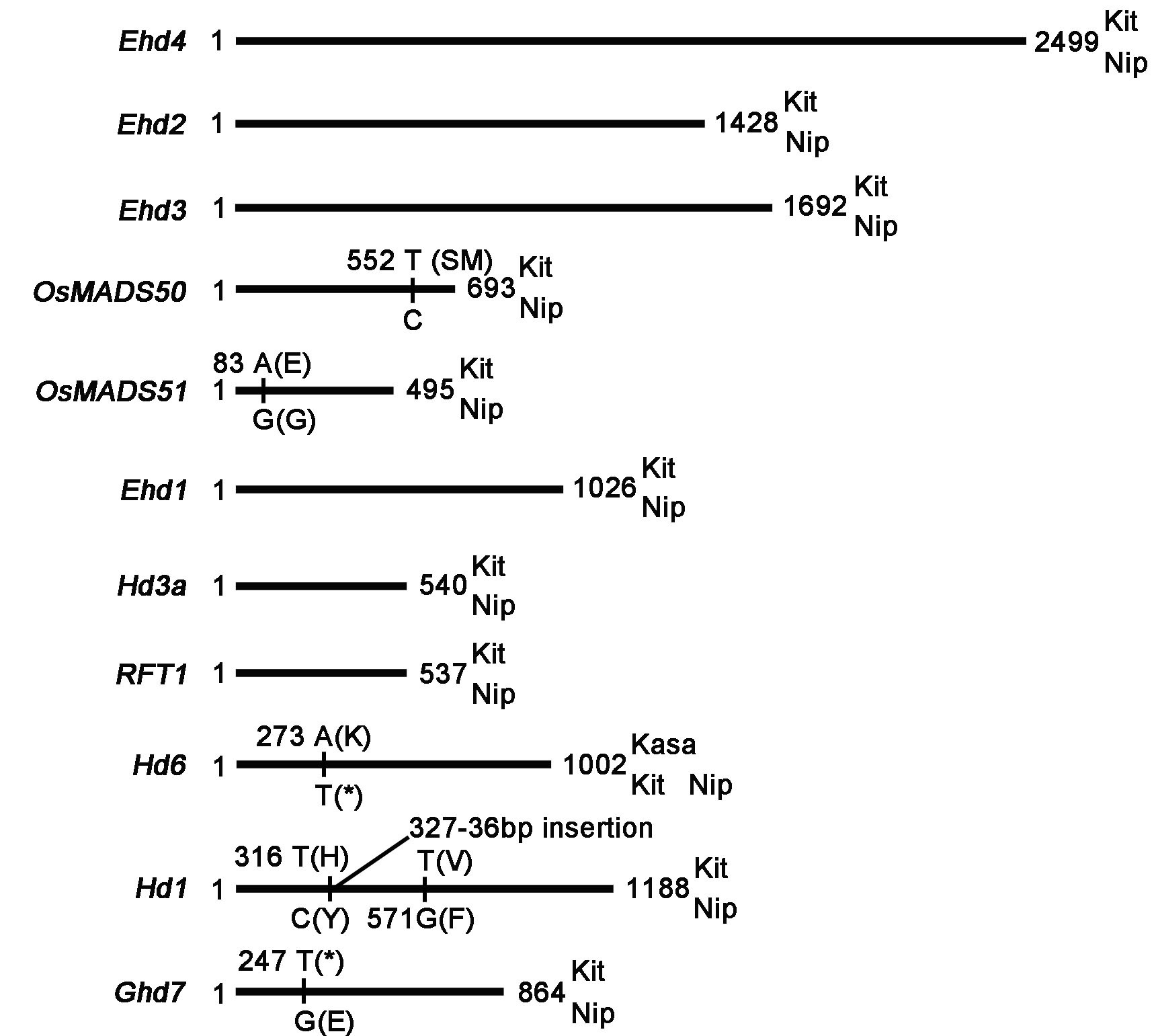

Supplement: Figure S1 — Alignment of the open reading frames of eleven flowering related genes between Kita-ake and Nipponbare. Kasa, Kasalash. SM, synonymous mutations; Asterisks represent the premature stop codon. (JPG) [file pgen.1003281.s001.jpg]

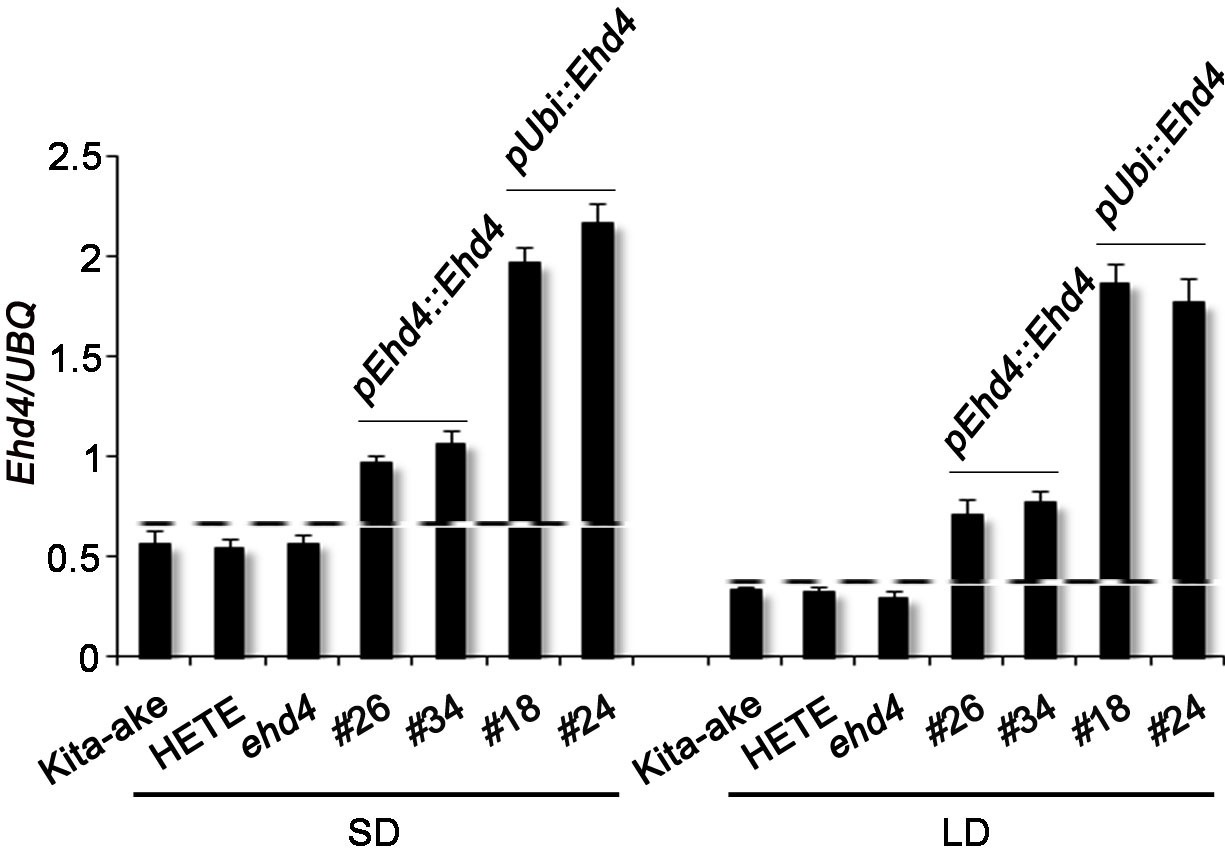

Supplement: Figure S2 — Transcript levels of Ehd4 in WT (Kita-ake), heterozygote (HETE), ehd4 and transgenic plants. Penultimate leaves were harvested at dawn from 28 d-old (SDs) and 35 d-old (LDs) plants. The rice Ubiquitin-1 (UBQ) gene was used as the internal control. Values are shown as mean±s.d (standard deviations) of three independent experiments and two biological replicates. (JPG) [file pgen.1003281.s002.jpg]

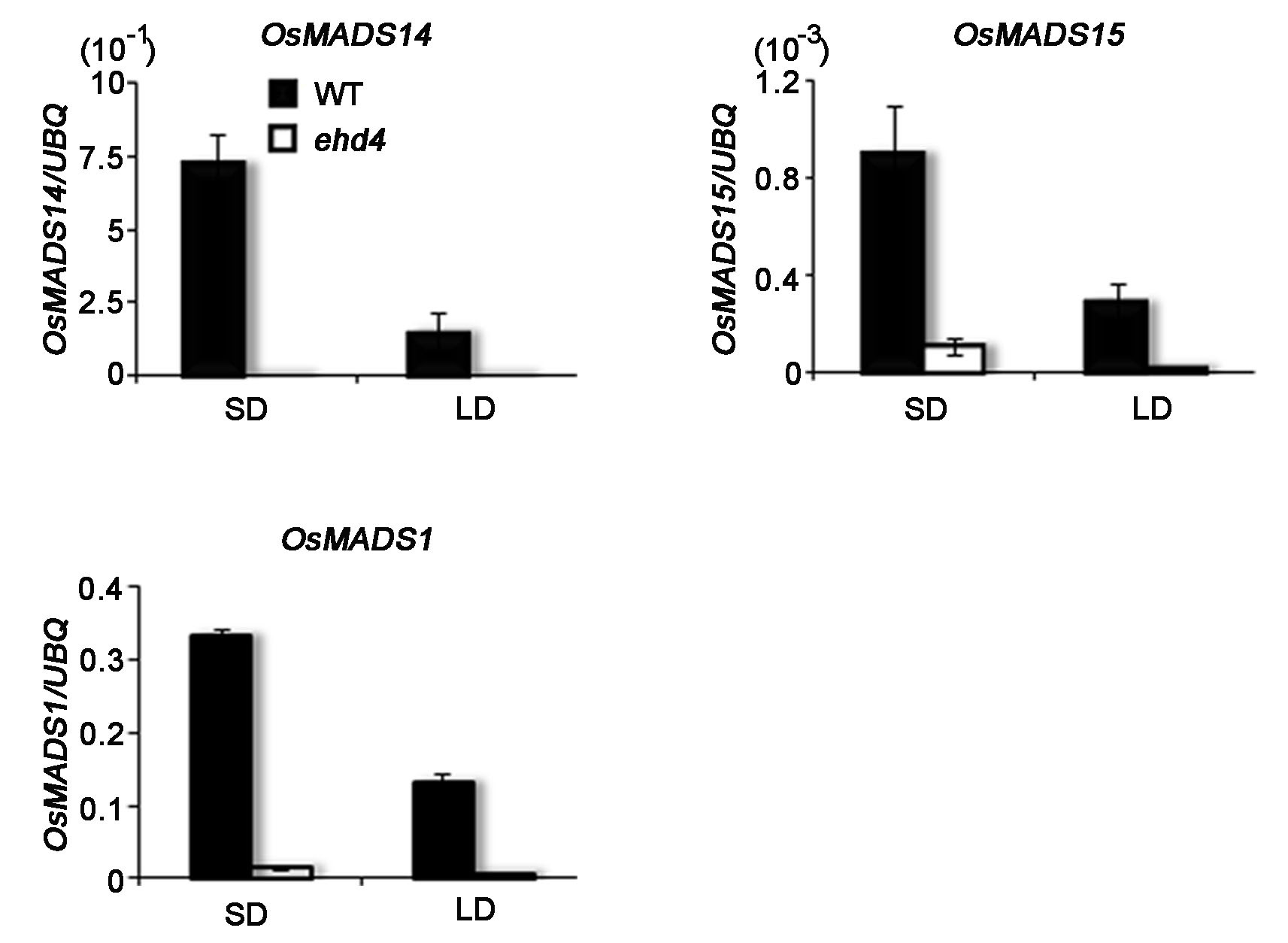

Supplement: Figure S3 — Transcript levels of OsMADS14, OsMADS15 and OsMADS1 in WT (Kita-ake) and ehd4 plants. Penultimate leaves were harvested around reported peak expression level of each gene during the 24 hrs photoperiod - at dawn from 28 d-old (SDs) and 35 d-old (LDs) plants. The rice Ubiquitin-1 (UBQ) gene was used as the internal control. Values are shown as mean±s.d (standard deviations) of three independent experiments and two biological replicates. (JPG) [file pgen.1003281.s003.jpg]

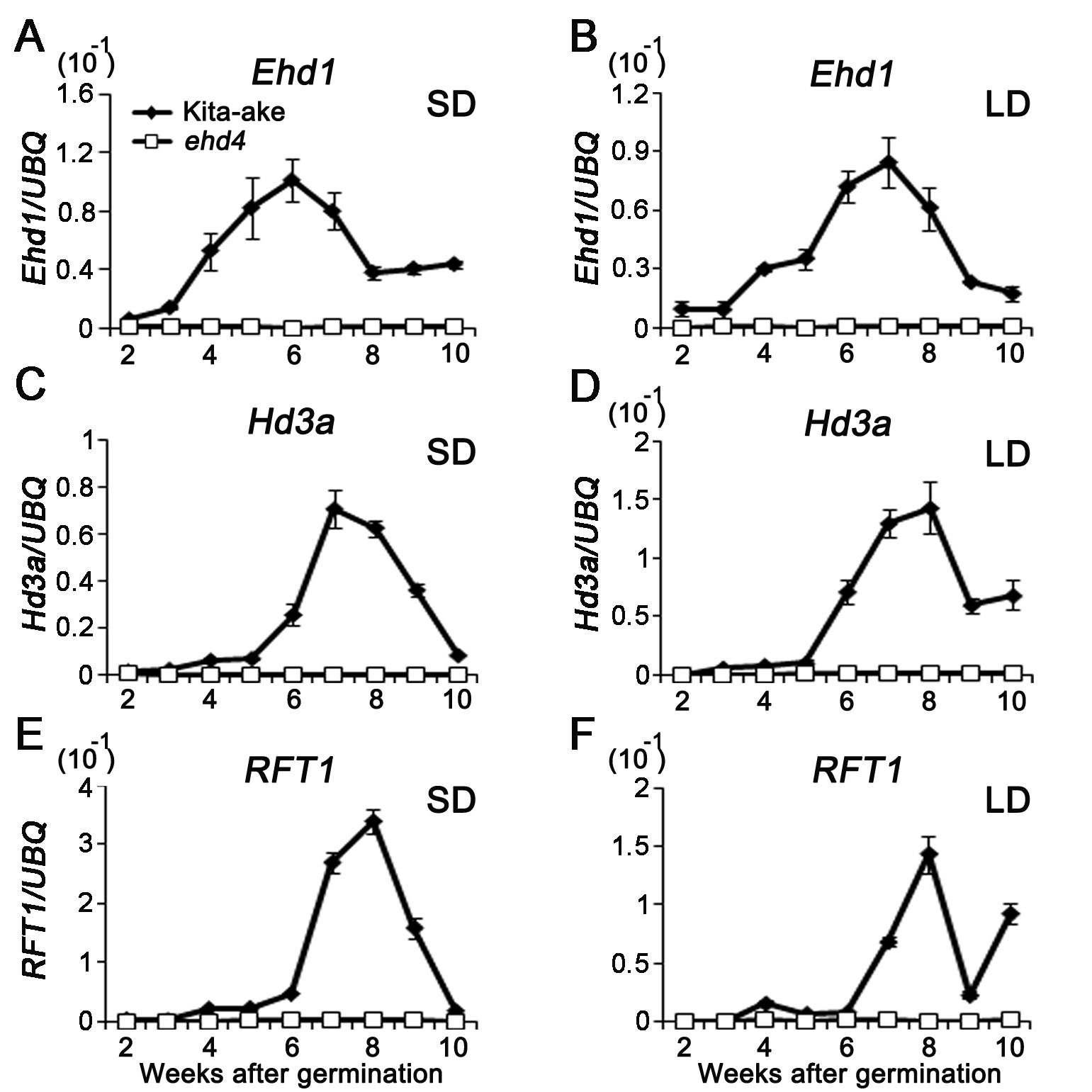

Supplement: Figure S4 — Developmental expression pattern of Ehd1, Hd3a and RFT1 in WT (Kita-ake) and ehd4 plants under SDs (A, C and E) and LDs (B, D and F). The rice Ubiquitin-1 (UBQ) gene was used as the internal control in the quantitative RT-PCR analysis. Values are shown as mean±s.d. (standard deviations) of three independent experiments and two biological replicates. (JPG) [file pgen.1003281.s004.jpg]

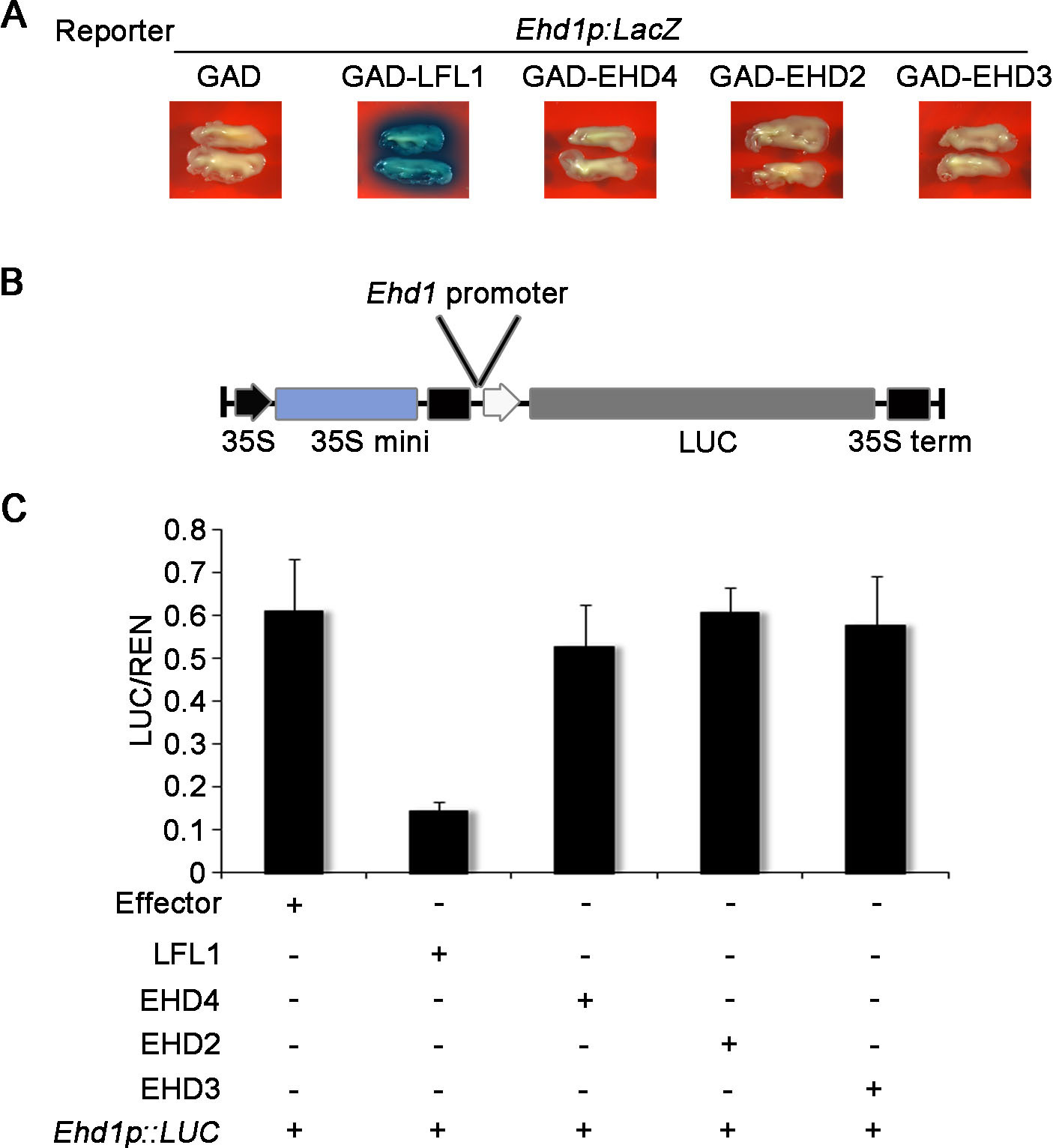

Supplement: Figure S5 — Yeast One-Hybrid and Bioluminescence assays. (A) GAD-LFL1 (positive control; [45]), but not GAD-EHD2, GAD-EHD3, GAD-EHD4 or GAD itself (negative control), strongly activate expression of the LacZ reporter genes driven by the Ehd1 promoter (3.2 kb upstream of the ATG start codon) in yeast one-hybrid assay. (B) Structure of the vector used for transient expression. 35S, 35S CaMV promoter; REN, renilla luciferase; 35S mini, 35S CaMV minimum promoter; LUC, luciferase gene; 35S term, 35S CaMV terminator. (C) Relative reporter activity (LUC/REN) in rice protoplasts. Bioluminescence assays showing that expression of Ehd1::LUC reporter was not induced by EHD4, EHD2, EHD3 or GAD (empty vector) itself but strongly repressed by LFL1 (positive control; [45]) in rice leaf protoplasts. The relative LUC activities normalized to the REN activity are shown (LUC/REN, n = 3). (JPG) [file pgen.1003281.s005.jpg]

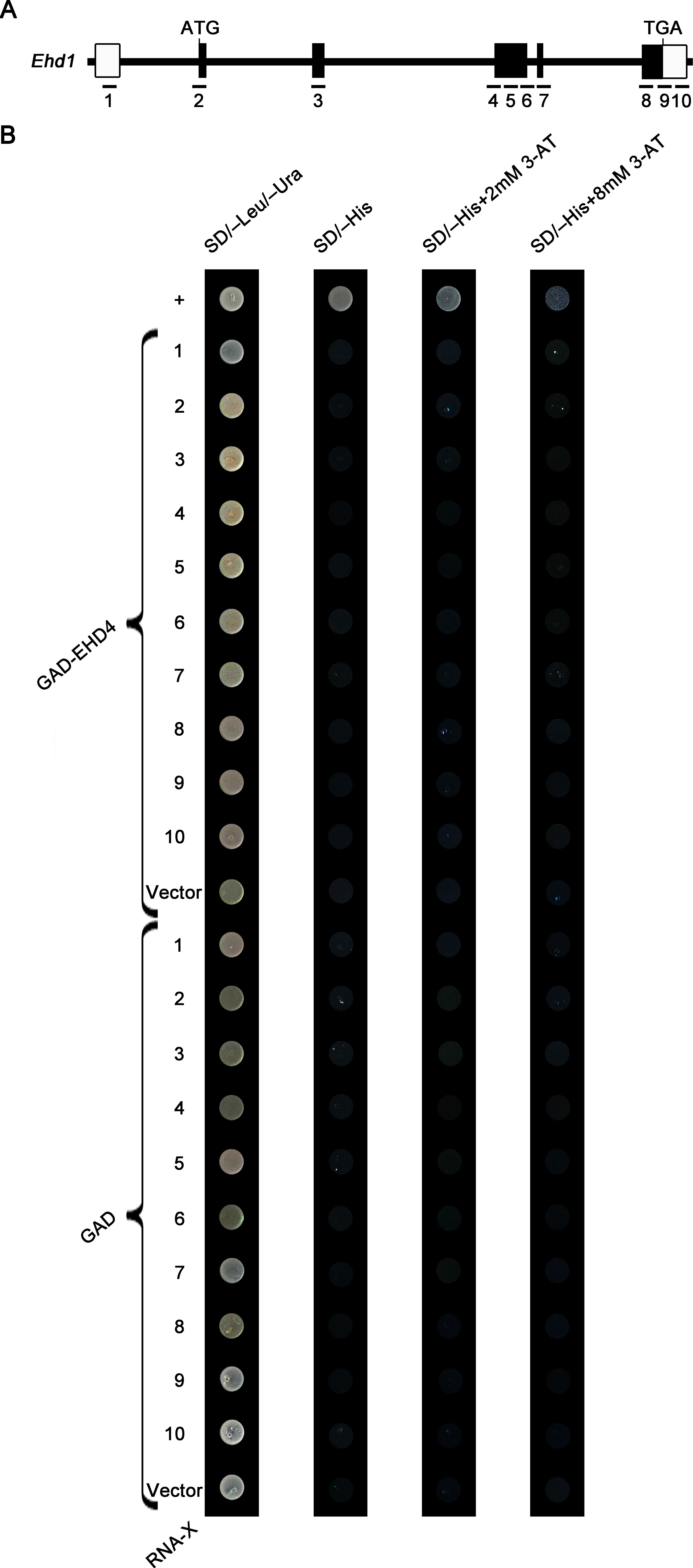

Supplement: Figure S6 — Yeast Three-Hybrid assay. (A) Genomic structure of the Ehd1 locus. Regions used for assays in (B) are underlined and numbered in order. (B) Yeast Three-Hybrid assays showing that EHD4 did not interact with any part of Ehd1 mRNA. The plasmid pIIIA/IRE-MS2 expressing 5′ IRE-MS2 3′ hybrid RNA from the yeast RNAseP promoter and the plasmid pAD-IRP expressing the rabbit Iron Regulatory Protein fused to the Gal 4 Activation Domain was used as the positive control [46] and the corresponding empty vectors were used as the negative control. (JPG) [file pgen.1003281.s006.jpg]

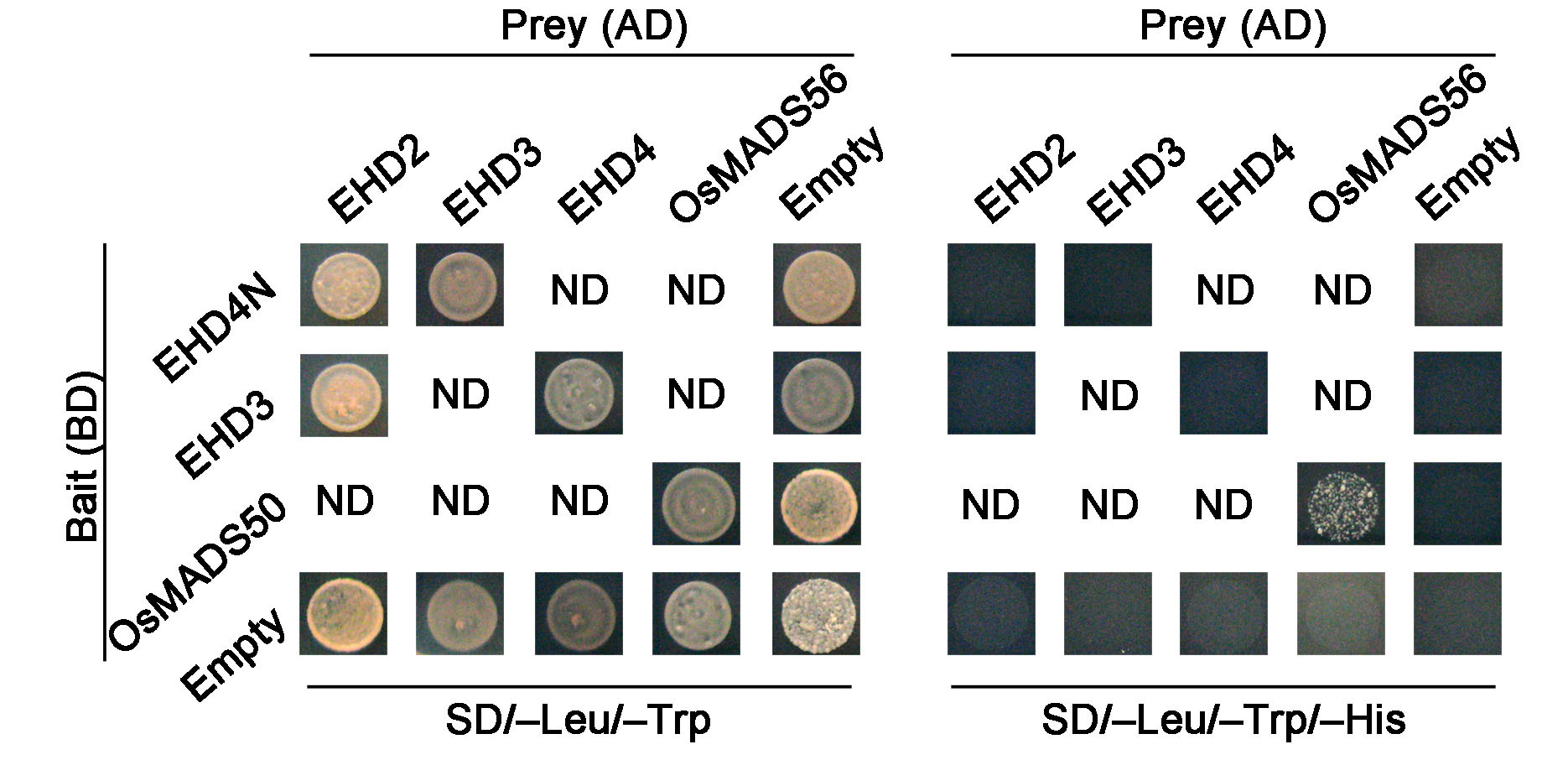

Supplement: Figure S7 — Yeast Two-Hybrid Assay. Yeast two-hybrid assays showing that EHD2, EHD3 and EHD4 did not interact with each other. BD-OsMADS50 and AD-OsMADS56 were used as the positive control [50] and the empty vector were used as the negative control. ND, not determined. (JPG) [file pgen.1003281.s007.jpg]

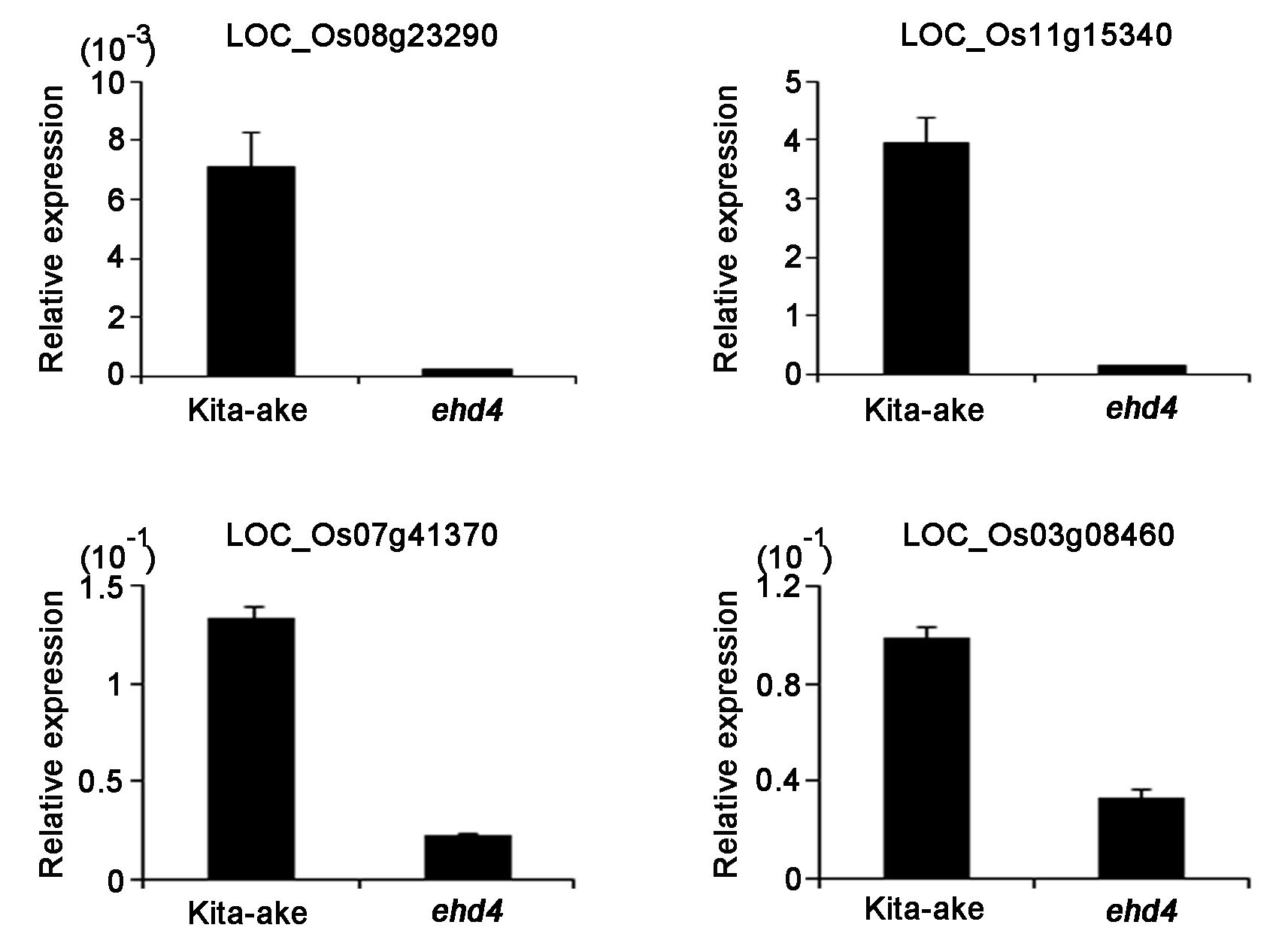

Supplement: Figure S8 — qRT-PCR confirmation of RNA-seq results. Four genes with reduced expression in ehd4 as determined by RNA-seq, were chosen for qRT-PCR assay. Independent penultimate leaves of 28 d-old plants grown under LDs were collected at dawn. The rice Ubiquitin-1 (UBQ) gene was used as the internal control. Values are shown as mean±s.d (standard deviations) of three independent experiments. (JPG) [file pgen.1003281.s008.jpg]

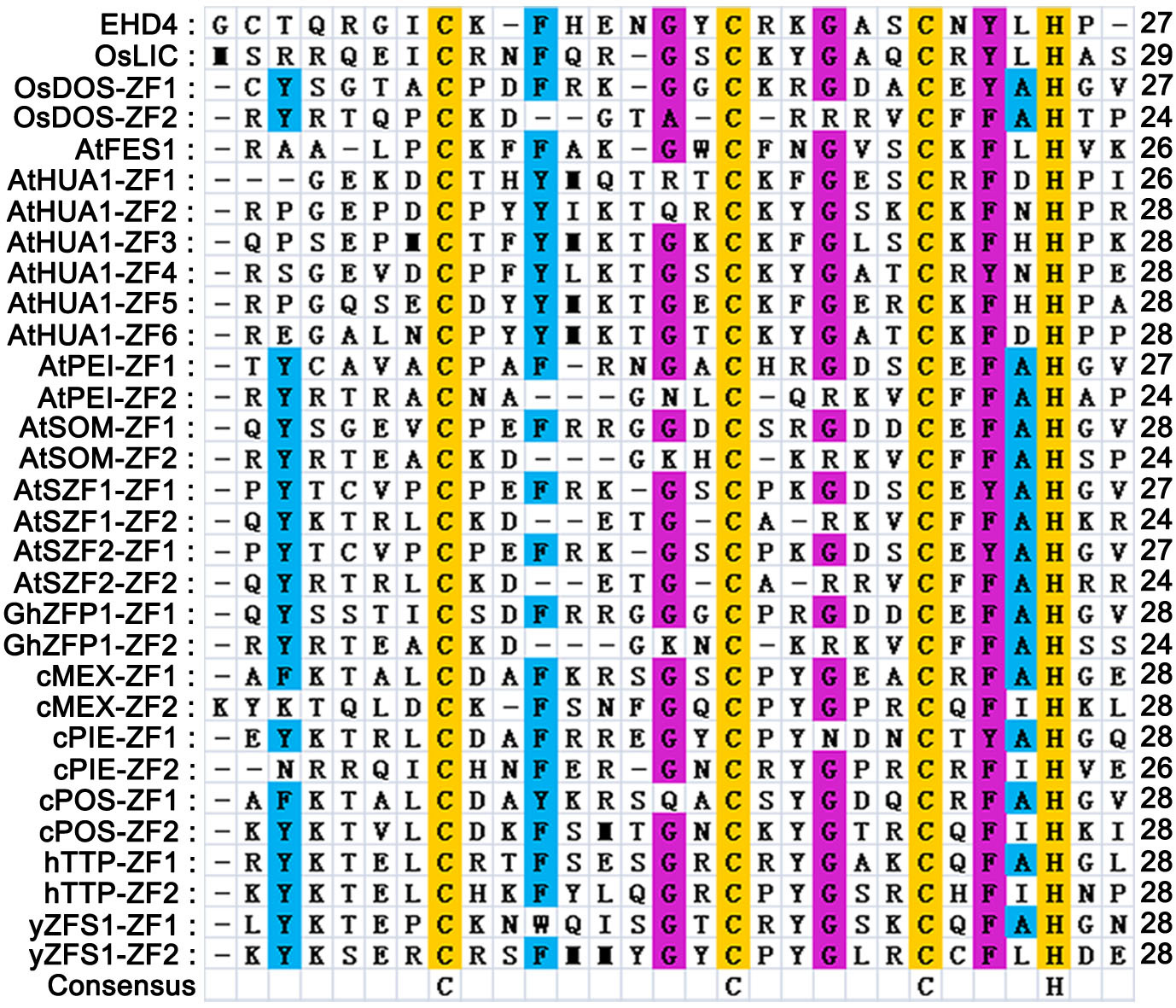

Supplement: Figure S9 — Alignment of the CCCH motif of EHD4 with the zinc fingers from other CCCH-type proteins. Zinc fingers are from rice OsLIC (Os06g49080) and OsDOS (Os01g09620), Arabidopsis FES1 (At2g33835), HUA1 (NP_187874), PEI (S22126), SOMNUS (At1g03790), SZF1 (At3g55980) and SZF2 (At2g40140), Cotton GhZFP1 (AY887895), C. elegans MEX-1 (U81043), PIE-1 (AAB17868) and POS-1 (T37246), human TTP (P26651) and yeast ZFS1(P47979). CCCH motifs from the same gene are shown as serial numbers. The consensus CCCH residues are shaded with yellow color. Other identical or similar residues are shaded with blue or purple color, respectively. (JPG) [file pgen.1003281.s009.jpg]

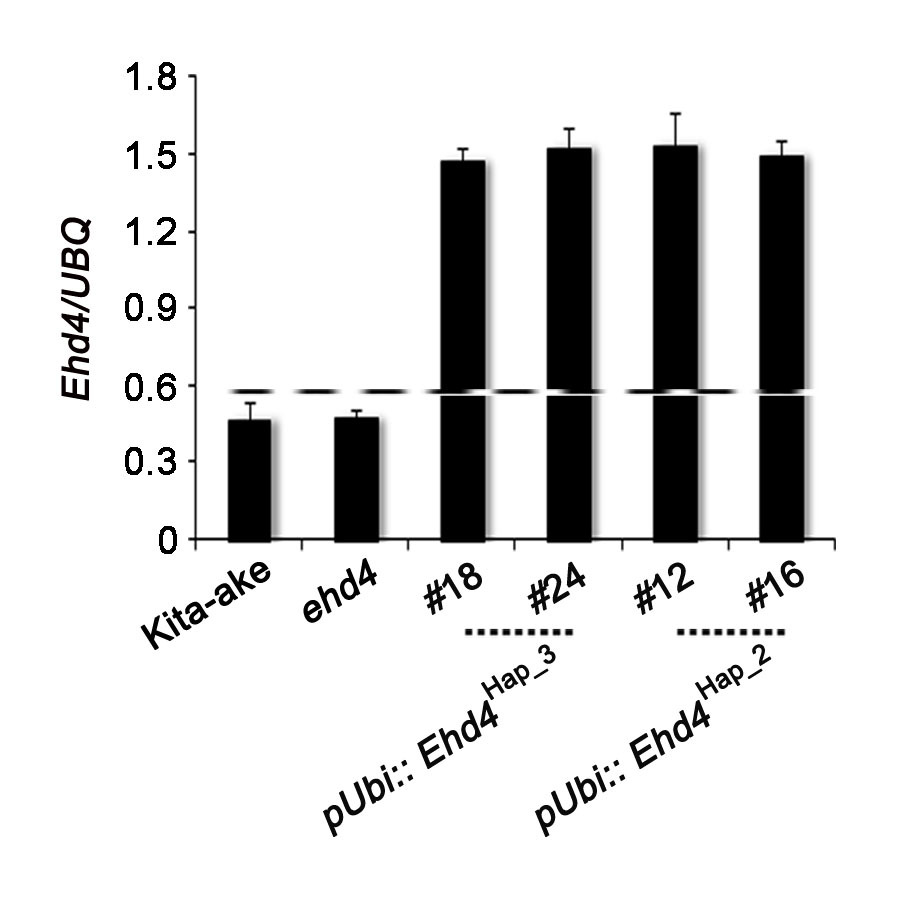

Supplement: Figure S10 — Transcript levels of Ehd4 in WT (Kita-ake), ehd4 and transgenic plants. Penultimate leaves were harvested at dawn from 35 d-old plants grown under natural long day conditions in Beijing. The rice Ubiquitin-1 (UBQ) gene was used as the internal control. Values are shown as mean±s.d (standard deviations) of three independent experiments and two biological replicates. (JPG) [file pgen.1003281.s010.jpg]
