# Supplementary material for: Ehd4 Encodes a Novel and Oryza-Genus-Specific Regulator of Photoperiodic Flowering in Rice
Source: PLoS Genet. 2013 Feb 21;9(2):e1003281. doi: 10.1371/journal.pgen.1003281 (PMC3578780; doi:10.1371/journal.pgen.1003281)
Supplement: Table S1 — Transcriptome analysis of ehd4 plants. Genes with expression level changes of at least 8 fold (ehd4/Wild Type) were listed. (DOC) [file pgen.1003281.s011.doc]

**Table S1.** Transcriptome analysis of *ehd4* plants. Genes with expression level changes of at least 8 fold (*ehd4*/Wild Type) were listed.

| **GeneID*** | **Log^2^ ratio** | **Annotation** |
| --- | --- | --- |
| **Down-regulated known flowering time-related genes** | | |
| LOC_Os10g32600 | -13.83 | *Ehd1* |
| LOC_Os06g06320 | -15.05 | *Hd3a* |
| LOC_Os06g06300 | -12.05 | *RFT1* |
| LOC_Os03g54160 | -11.63 | *OsMADS14* |
| LOC_Os07g01820 | -3.72 | *OsMADS15* |
| LOC_Os03g11614 | -4.52 | *OsMADS1* |
| LOC_Os01g51610 | -7.01 | *OsLFL1* |
| **Down-regulated TFs:** | | |
| **MADS box** | | |
| LOC_Os06g45650 | -9.59 | OsMADS30-MADS-box family gene with MIKCc type-box |
| LOC_Os12g21880 | -9.01 | OsMADS74-MADS-box family gene with M-alpha type-box |
| LOC_Os07g04170 | -7.87 | OsMADS90-MADS-box family gene with M-beta type-box |
| LOC_Os02g45770 | -7.25 | OsMADS6-MADS-box family gene with MIKCc type-box |
| LOC_Os07g41370 | -5.8 | OsMADS18-MADS-box family gene with MIKCc type-box |
| LOC_Os02g49840 | -3.63 | OsMADS57-MADS-box family gene with MIKCc type-box |
| **Zinc finger** | | |
| LOC_Os01g55340 | -3.79 | Dof zinc finger domain containing protein |
| LOC_Os01g10580 | -3.44 | B-box zinc finger family protein |
| LOC_Os07g48680 | -3.15 | Zinc finger, C3HC4 type domain containing protein, expressed |
| **MYB** | | |
| LOC_Os02g07170 | -14.19 | MYB family transcription factor |
| LOC_Os12g37690 | -12.12 | MYB family transcription factor |
| LOC_Os04g56990 | -3.44 | MYB-like DNA-binding domain containing protein |
| **WRKY** | | |
| LOC_Os01g53260 | -11.56 | OsWRKY23-Superfamily of TFs having WRKY and zinc finger domains |
| LOC_Os11g02520 | -3.54 | WRKY DNA-binding domain containing protein |
| **SBP** | | |
| LOC_Os07g32170 | -8.88 | OsSPL13 - SBP-box gene family member, expressed |
| **B3** | | |
| LOC_Os03g42370 | -8.29 | B3 DNA binding domain containing protein |
| LOC_Os03g08620 | -8.08 | B3 DNA binding domain containing protein, expressed |
| LOC_Os03g42410 | -7.71 | B3 DNA binding domain containing protein |
| LOC_Os01g52514 | -7.2 | B3 DNA binding domain containing protein, expressed |
| LOC_Os03g42230 | -3.68 | B3 DNA binding domain containing protein, expressed |
| **Others** | | |
| **Down-regulated** | | |
| LOC_Os01g62260 | -16.29 | thaumatin, putative, expressed |
| LOC_Os07g08240 | -15.75 | ZmEBE-1 protein, putative, expressed |
| LOC_Os12g25090 | -15.57 | expressed protein |
| LOC_Os03g17870 | -15 | metallothionein, putative, expressed |
| LOC_Os04g10940 | -14.9 | expressed protein |
| LOC_Os07g08150 | -14.75 | early light-induced protein, chloroplast precursor, putative, expressed |
| LOC_Os07g08160 | -14.32 | early light-induced protein, chloroplast precursor, putative, expressed |
| LOC_Os09g18050 | -13.69 | conserved hypothetical protein |
| LOC_Os04g56430 | -13.47 | cysteine-rich receptor-like protein kinase, putative, expressed |
| LOC_Os02g15930 | -13.21 | expressed protein |
| LOC_Os06g36560 | -13.09 | inositol oxygenase, putative, expressed |
| LOC_Os03g46060 | -12.85 | thaumatin family domain containing protein, expressed |
| LOC_Os05g39770 | -12.76 | aminotransferase, putative, expressed |
| LOC_Os08g39730 | -12.71 | cytochrome P450, putative, expressed |
| LOC_Os11g28940 | -12.65 | expressed protein |
| LOC_Os01g37200 | -12.5 | retrotransposon protein, putative, Ty3-gypsy subclass, expressed |
| LOC_Os04g59630 | -12.26 | prenylcysteine oxidase 1 precursor, putative, expressed |
| LOC_Os05g49940 | -12.12 | expressed protein |
| LOC_Os08g30014 | -12.08 | hypothetical protein |
| LOC_Os11g47500 | -12.04 | glycosyl hydrolase, putative, expressed |
| LOC_Os04g27670 | -11.92 | terpene synthase family, metal binding domain containing protein, expressed |
| LOC_Os07g25960 | -11.84 | retrotransposon protein, putative, unclassified, expressed |
| LOC_Os03g04720 | -11.79 | retrotransposon protein, putative, unclassified |
| LOC_Os11g15340 | -11.73 | SAM dependent carboxyl methyltransferase family protein, putative, expressed |
| LOC_Os01g05010 | -11.6 | mitochondrial glycoprotein, putative, expressed |
| LOC_Os08g02030 | -11.58 | transferase family protein, putative, expressed |
| LOC_Os12g12854 | -11.5 | expressed protein |
| LOC_Os10g20450 | -11.46 | MATE efflux family protein, putative, expressed |
| LOC_Os09g27260 | -11.12 | plant viral response family protein, putative, expressed |
| LOC_Os03g06250 | -11.08 | retrotransposon protein, putative, unclassified, expressed |
| LOC_Os02g48570 | -10.81 | peptide transporter PTR2, putative, expressed |
| LOC_Os02g10880 | -10.77 | UDP-glucoronosyl and UDP-glucosyl transferase domain containing protein, expressed |
| LOC_Os08g18974 | -10.76 | expressed protein |
| LOC_Os09g18030 | -10.7 | Leucine Rich Repeat family protein |
| LOC_Os03g16920 | -10.6 | DnaK family protein, putative, expressed |
| LOC_Os07g34520 | -10.56 | isocitrate lyase, putative, expressed |
| LOC_Os12g42910 | -10.47 | sodium/calcium exchanger protein, putative, expressed |
| LOC_Os06g29220 | -10.41 | erythronate-4-phosphate dehydrogenase domain containing protein, expressed |
| LOC_Os05g02450 | -10.37 | expressed protein |
| LOC_Os08g23290 | -10.33 | serine/threonine-protein kinase BRI1-like 2 precursor, putative |
| LOC_Os02g36210 | -9.99 | ent-kaurene synthase, chloroplast precursor, putative, expressed |
| LOC_Os04g06590 | -9.87 | expressed protein |
| LOC_Os06g10560 | -9.74 | leaf senescence related protein, putative, expressed |
| LOC_Os01g72990 | -9.6 | expressed protein |
| LOC_Os09g16995 | -9.58 | transposon protein, putative, CACTA, En/Spm sub-class, expressed |
| LOC_Os09g28370 | -8.5 | retrotransposon protein, putative, unclassified |
| LOC_Os11g19864 | -6.84 | transposon protein, putative, Pong sub-class, expressed |
| LOC_Os03g43750 | -6.48 | retrotransposon, putative, centromere-specific, expressed |
| LOC_Os06g16640 | -6.29 | carboxyl-terminal peptidase, putative, expressed |
| LOC_Os01g14410 | -6.15 | early light-induced protein, chloroplast precursor, putative, expressed |
| LOC_Os04g03050 | -6.09 | OsSub34-Putative Subtilisin homologue, expressed |
| LOC_Os01g47300 | -6.07 | retrotransposon protein, putative, unclassified, expressed |
| LOC_Os07g23640 | -6.03 | retrotransposon protein, putative, Ty3-gypsy subclass, expressed |
| LOC_Os02g51930 | -5.74 | cytokinin-O-glucosyltransferase 2, putative, expressed |
| LOC_Os03g04080 | -5.63 | expressed protein |
| LOC_Os11g19850 | -5.48 | hypothetical protein |
| LOC_Os12g33130 | -5.43 | expressed protein |
| LOC_Os05g12320 | -5.41 | nodulin MtN3 family protein, putative, expressed |
| LOC_Os01g45640 | -5.37 | tat pathway signal sequence family protein, expressed |
| LOC_Os04g38680 | -5.3 | transmembrane amino acid transporter protein, putative, expressed |
| LOC_Os03g17460 | -5.23 | IN2-1 protein, putative, expressed |
| LOC_Os01g06740 | -5.19 | ribosome inactivating protein, putative, expressed |
| LOC_Os03g18130 | -5.14 | asparagine synthetase, putative, expressed |
| LOC_Os03g30300 | -5.11 | 6-phosphogluconolactonase, putative, expressed |
| LOC_Os01g42860 | -5.11 | inhibitor I family protein, putative, expressed |
| LOC_Os01g64120 | -4.98 | 2Fe-2S iron-sulfur cluster binding domain containing protein, expressed |
| LOC_Os03g29190 | -4.94 | PDI, putative, expressed |
| LOC_Os02g54254 | -4.85 | saccharopine dehydrogenase, putative, expressed |
| LOC_Os08g07720 | -4.84 | transferase family protein, putative, expressed |
| LOC_Os10g26940 | -4.82 | BURP domain containing protein, expressed |
| LOC_Os05g48810 | -4.74 | dnaJ domain containing protein, expressed |
| LOC_Os11g07225 | -4.63 | conserved hypothetical protein |
| LOC_Os05g37830 | -4.57 | expressed protein |
| LOC_Os02g16670 | -4.51 | ATP-binding domain-containing protein, putative, expressed |
| LOC_Os09g19229 | -4.48 | protein kinase domain containing protein, expressed |
| LOC_Os04g59190 | -4.46 | peroxidase precursor, putative, expressed |
| LOC_Os01g11940 | -4.44 | OsFTL1 FT-Like1 homologous to Flowering Locus T gene |
| LOC_Os07g26110 | -4.44 | membrane associated DUF588 domain containing protein, putative, expressed |
| LOC_Os12g37519 | -4.43 | retrotransposon protein, putative, unclassified, expressed |
| LOC_Os04g49780 | -4.38 | transposon protein, putative, CACTA, En/Spm sub-class |
| LOC_Os02g34960 | -4.38 | pentatricopeptide, putative, expressed |
| LOC_Os03g55330 | -4.38 | ankyrin repeat domain containing protein, putative, expressed |
| LOC_Os01g71624 | -4.34 | expressed protein |
| LOC_Os09g10054 | -4.32 | disease resistance protein RPS2, putative, expressed |
| LOC_Os07g48450 | -4.31 | no apical meristem protein, putative, expressed |
| LOC_Os06g46740 | -4.31 | early nodulin 20 precursor, putative, expressed |
| LOC_Os02g40330 | -4.27 | retrotransposon protein, putative, Ty3-gypsy subclass, expressed |
| LOC_Os06g15910 | -4.25 | potassium transporter, putative, expressed |
| LOC_Os04g43916 | -4.23 | expressed protein |
| LOC_Os08g16050 | -4.23 | tetraspanin family protein, putative, expressed |
| LOC_Os10g38160 | -4.23 | glutathione S-transferase, putative, expressed |
| LOC_Os02g01710 | -4.23 | peptidase family C78 domain containing protein, expressed |
| LOC_Os04g10924 | -4.19 | hypothetical protein |
| LOC_Os03g17790 | -4.17 | OsRCI2-5 - Putative low temperature and salt responsive protein, expressed |
| LOC_Os10g37340 | -4.16 | cystathionine gamma-synthase, putative, expressed |
| LOC_Os08g13440 | -4.15 | cupin domain containing protein, expressed |
| LOC_Os07g12320 | -4.15 | WD domain, G-beta repeat domain containing protein, expressed |
| LOC_Os05g42960 | -4.15 | retrotransposon protein, putative, unclassified, expressed |
| LOC_Os01g62290 | -4.15 | DnaK family protein, putative, expressed |
| LOC_Os06g47210 | -4.15 | oligopeptidase, putative, expressed |
| LOC_Os11g06980 | -4.11 | BURP domain containing protein, expressed |
| LOC_Os07g27390 | -4.09 | retrotransposon protein, putative, unclassified, expressed |
| LOC_Os12g25720 | -4.07 | expressed protein |
| LOC_Os05g45070 | -4.07 | harpin-induced protein 1 domain containing protein, expressed |
| LOC_Os02g45040 | -4.05 | retrotransposon protein, putative, unclassified, expressed |
| LOC_Os10g31864 | -4.05 | conserved hypothetical protein |
| LOC_Os02g33030 | -4.01 | bifunctional monodehydroascorbate reductase and carbonic anhydrasenectarin-3 precursor, putative, expressed |
| LOC_Os01g74490 | -3.99 | heavy metal-associated domain containing protein, expressed |
| LOC_Os05g33890 | -3.98 | microtubule associated protein, putative, expressed |
| LOC_Os08g07290 | -3.98 | HEAT repeat family protein, putative, expressed |
| LOC_Os07g24830 | -3.98 | thionin-like peptide, putative, expressed |
| LOC_Os04g41140 | -3.98 | PPR repeat containing protein, expressed |
| LOC_Os09g18230 | -3.97 | expressed protein |
| LOC_Os05g38720 | -3.94 | Lung seven transmembrane receptor domain containing protein, putative, expressed |
| LOC_Os05g16430 | -3.91 | SHR5-receptor-like kinase, putative, expressed |
| LOC_Os11g02369 | -3.89 | LTPL7 - Protease inhibitor/seed storage/LTP family protein precursor, expressed |
| LOC_Os06g48210 | -3.89 | DEAD-box ATP-dependent RNA helicase, putative, expressed |
| LOC_Os04g20320 | -3.89 | retrotransposon protein, putative, unclassified |
| LOC_Os03g02874 | -3.87 | retrotransposon protein, putative, Ty3-gypsy subclass, expressed |
| LOC_Os07g45074 | -3.79 | FAT domain-containing protein, putative, expressed |
| LOC_Os01g06660 | -3.79 | thiamine pyrophosphate enzyme, C-terminal TPP binding domain containing protein, expressed |
| LOC_Os02g51890 | -3.79 | RNA recognition motif containing protein, putative, expressed |
| LOC_Os07g05365 | -3.77 | photosystem II 10 kDa polypeptide, chloroplast precursor, putative, expressed |
| LOC_Os04g51090 | -3.76 | tRNA-splicing endonuclease positive effector-related, putative, expressed |
| LOC_Os06g45184 | -3.76 | retrotransposon protein, putative, Ty3-gypsy subclass, expressed |
| LOC_Os07g23570 | -3.68 | cytochrome P450 72A1, putative, expressed |
| LOC_Os05g38264 | -3.65 | expressed protein |
| LOC_Os01g62610 | -3.64 | peptidyl-prolyl cis-trans isomerase, FKBP-type, putative, expressed |
| LOC_Os07g41190 | -3.63 | WD domain, G-beta repeat domain containing protein, expressed |
| LOC_Os09g15050 | -3.6 | ent-kaurene synthase A, chloroplast precursor, putative, expressed |
| LOC_Os08g36040 | -3.57 | plant viral response family protein, putative, expressed |
| LOC_Os04g56250 | -3.54 | OsFBX152 - F-box domain containing protein, expressed |
| LOC_Os03g57200 | -3.54 | glutathione S-transferase, putative, expressed |
| LOC_Os06g03520 | -3.54 | DUF581 domain containing protein, expressed |
| LOC_Os05g37350 | -3.51 | hhH-GPD superfamily base excision DNA repair protein |
| LOC_Os02g40784 | -3.49 | WAX2, putative, expressed |
| LOC_Os01g32780 | -3.48 | universal stress protein domain containing protein, putative, expressed |
| LOC_Os07g43604 | -3.48 | hypothetical protein |
| LOC_Os12g16720 | -3.44 | cytochrome P450 71A1, putative, expressed |
| LOC_Os01g58130 | -3.44 | expressed protein |
| LOC_Os06g05470 | -3.44 | expressed protein |
| LOC_Os06g14406 | -3.39 | SYD, putative, expressed |
| LOC_Os08g01290 | -3.38 | OsFBT12 - F-box and tubby domain containing protein, expressed |
| LOC_Os03g19462 | -3.35 | conserved hypothetical protein |
| LOC_Os01g21160 | -3.35 | 2-oxo acid dehydrogenases acyltransferase domain containing protein, expressed |
| LOC_Os04g46940 | -3.33 | copper-transporting ATPase 3, putative, expressed |
| LOC_Os07g44140 | -3.32 | cytochrome P450 72A1, putative, expressed |
| LOC_Os06g01360 | -3.31 | homogentisate 1,2-dioxygenase, putative, expressed |
| LOC_Os03g16030 | -3.31 | hsp20/alpha crystallin family protein, putative, expressed |
| LOC_Os01g52070 | -3.31 | potassium channel AKT1, putative, expressed |
| LOC_Os04g58070 | -3.31 | aspartic proteinase nepenthesin precursor, putative, expressed |
| LOC_Os03g12820 | -3.28 | ATP8, putative, expressed |
| LOC_Os12g41830 | -3.27 | Methyltransferase small domain containing protein, expressed |
| LOC_Os05g16420 | -3.26 | SHR5-receptor-like kinase, putative, expressed |
| LOC_Os09g34250 | -3.25 | UDP-glucoronosyl and UDP-glucosyl transferase domain containing protein, expressed |
| LOC_Os09g19300 | -3.23 | retrotransposon protein, putative, unclassified, expressed |
| LOC_Os02g53410 | -3.23 | expressed protein |
| LOC_Os11g35840 | -3.23 | conserved hypothetical protein |
| LOC_Os08g39660 | -3.23 | cytochrome P450, putative, expressed |
| LOC_Os10g27040 | -3.18 | retrotransposon protein, putative, unclassified |
| LOC_Os05g09500 | -3.15 | hexokinase, putative, expressed |
| LOC_Os07g44110 | -3.15 | cytochrome P450 72A1, putative, expressed |
| LOC_Os01g42410 | -3.15 | pleiotropic drug resistance protein, putative, expressed |
| LOC_Os03g08460 | -3.15 | AP2 domain containing protein, expressed |
| **Up-regulated** | | |
| LOC_Os07g05840 | 16.51 | expressed protein |
| LOC_Os11g35300 | 15.77 | conserved hypothetical protein |
| LOC_Os07g05860 | 15.44 | hypothetical protein |
| LOC_Os07g05850 | 15.26 | transposon protein, putative, CACTA, En/Spm sub-class |
| LOC_Os06g05230 | 15.26 | retrotransposon protein, putative, unclassified |
| LOC_Os03g51530 | 14.13 | expressed protein |
| LOC_Os03g02470 | 13.88 | expressed protein |
| LOC_Os10g24170 | 13.44 | retrotransposon protein, putative, unclassified |
| LOC_Os06g15430 | 13.31 | expressed protein |
| LOC_Os03g08520 | 13.04 | DUF581 domain containing protein, expressed |
| LOC_Os01g03360 | 12.93 | BBTI5 - Bowman-Birk type bran trypsin inhibitor precursor, expressed |
| LOC_Os07g23510 | 12.9 | hypothetical protein |
| LOC_Os11g41780 | 12.89 | transposon protein, putative, CACTA, En/Spm sub-class |
| LOC_Os11g45040 | 12.88 | hypothetical protein |
| LOC_Os09g36700 | 12.54 | ribonuclease T2 family domain containing protein, expressed |
| LOC_Os10g11889 | 12.53 | expressed protein |
| LOC_Os03g18850 | 12.32 | pathogenesis-related Bet v I family protein, putative, expressed |
| LOC_Os07g32710 | 12.17 | retrotransposon protein, putative, unclassified, expressed |
| LOC_Os06g19095 | 12.06 | expressed protein |
| LOC_Os05g12400 | 12.05 | BURP domain containing protein, expressed |
| LOC_Os11g41770 | 11.82 | conserved hypothetical protein |
| LOC_Os03g43100 | 11.8 | expressed protein |
| LOC_Os05g36240 | 11.52 | expressed protein |
| LOC_Os11g17540 | 11.09 | retrotransposon protein, putative, Ty1-copia subclass, expressed |
| LOC_Os12g24020 | 10.84 | rhodanese-like domain containing protein, putative, expressed |
| LOC_Os01g06750 | 10.74 | verticillium wilt disease resistance protein precursor, putative |
| LOC_Os12g13910 | 10.66 | expressed protein |
| LOC_Os11g41680 | 10.62 | cytochrome P450, putative, expressed |
| LOC_Os11g17380 | 10.25 | protein kinase domain containing protein |
| LOC_Os06g39840 | 10.21 | retrotransposon protein, putative, unclassified |
| LOC_Os01g03310 | 5.9 | BBTI1 - Bowman-Birk type bran trypsin inhibitor precursor, expressed |
| LOC_Os01g45914 | 5.74 | expressed protein |
| LOC_Os08g41880 | 5.23 | nucleotide pyrophosphatase/phosphodiesterase, putative, expressed |
| LOC_Os06g37690 | 4.98 | S-locus-like receptor protein kinase, putative |
| LOC_Os03g08330 | 4.97 | ZIM domain containing protein, putative, expressed |
| LOC_Os02g26210 | 4.92 | flowering promoting factor-like 1, putative, expressed |
| LOC_Os08g36860 | 4.91 | cytochrome P450, putative, expressed |
| LOC_Os07g03040 | 4.68 | expressed protein |
| LOC_Os05g10310 | 4.67 | acid phosphatase, putative, expressed |
| LOC_Os12g38170 | 4.65 | osmotin, putative, expressed |
| LOC_Os01g12210 | 4.24 | aluminum-activated malate transporter, putative, expressed |
| LOC_Os08g37040 | 4.19 | gibberellin receptor GID1L2, putative, expressed |
| LOC_Os06g29270 | 4.15 | retrotransposon protein, putative, unclassified |
| LOC_Os05g15770 | 4.14 | glycosyl hydrolase, putative, expressed |
| LOC_Os11g04720 | 4.07 | OsRR10 type-A response regulator, expressed |
| LOC_Os01g41420 | 4.06 | transmembrane amino acid transporter protein, putative, expressed |
| LOC_Os01g54620 | 4.04 | CESA4 - cellulose synthase, expressed |
| LOC_Os03g63330 | 3.86 | aspartokinase, chloroplast precursor, putative, expressed |
| LOC_Os09g26670 | 3.82 | expressed protein |
| LOC_Os03g22634 | 3.79 | terpene synthase, putative, expressed |
| LOC_Os05g06920 | 3.76 | relA-SpoT like protein RSH4, putative, expressed |
| LOC_Os01g13690 | 3.65 | ligA, putative, expressed |
| LOC_Os07g27880 | 3.6 | O-methyltransferase, putative |
| LOC_Os06g39110 | 3.53 | expressed protein |
| LOC_Os07g05360 | 3.48 | photosystem II 10 kDa polypeptide, chloroplast precursor, putative, expressed |
| LOC_Os11g45050 | 3.43 | NBS-LRR disease resistance protein, putative, expressed |
| LOC_Os09g25490 | 3.41 | CESA9 - cellulose synthase, expressed |
| LOC_Os01g08140 | 3.39 | phototropic-responsive NPH3 family protein, putative |
| LOC_Os05g32420 | 3.37 | expressed protein |
| LOC_Os07g33450 | 3.35 | retrotransposon protein, putative, unclassified |
| LOC_Os10g25290 | 3.26 | ZIM domain containing protein, putative, expressed |
| LOC_Os06g15730 | 3.18 | expressed protein |
| LOC_Os06g21140 | 3.16 | glycine-rich cell wall structural protein precursor, putative, expressed |
| LOC_Os02g03710 | 3.16 | UP-9A, putative, expressed |
| LOC_Os03g53540 | 3.16 | expressed protein |

**P*-value of differential gene expression test was less than 0.05.
