# Supplementary material for: Ehd4 Encodes a Novel and Oryza-Genus-Specific Regulator of Photoperiodic Flowering in Rice
Source: PLoS Genet. 2013 Feb 21;9(2):e1003281. doi: 10.1371/journal.pgen.1003281 (PMC3578780; doi:10.1371/journal.pgen.1003281)
Supplement: Table S2 — Summary of the Ehd4 allele types in cultivated and wild rice. (DOC) [file pgen.1003281.s012.doc]

**Table S2.** Summary of the *Ehd4* allele type in cultivated and wild rice.

| Variety | Species | Subspecies | Accession No. | Origin | Identity (%) | Allele type |
| --- | --- | --- | --- | --- | --- | --- |
| Kita-ake | *O. sativa* | Japonica^a^ | N/A | Japan | 100 | Hap3 |
| Nipponbare | *O. sativa* | Japonica^a^ | N/A | Japan | 100 | Hap3 |
| Jinbaoyin | *O. sativa* | Japonica^a^ | 13-00723 | China | 99.4 | Hap1 |
| Lengshuigu2 | *O. sativa* | Japonica^a^ | 21-01970 | China | 100 | Hap3 |
| Xishen15 | *O. sativa* | Japonica^a^ | 15-04286 | China | 100 | Hap3 |
| Yuyannuo | *O. sativa* | Japonica^a^ | 21-02235 | China | 100 | Hap3 |
| Baihe | *O. sativa* | Japonica^a^ | 22-03892 | China | 100 | Hap3 |
| Yajiashu | *O. sativa* | Japonica^a^ | 13-00940 | China | 100 | Hap3 |
| Lengshuinuo | *O. sativa* | Japonica^a^ | 21-01989 | China | 100 | Hap3 |
| Wudijing | *O. sativa* | Japonica^a^ | 13-00606 | China | 99.5 | Hap2 |
| Sanlicun | *O. sativa* | Japonica^a^ | 15-04016 | China | 99.5 | Hap2 |
| Taidongludao | *O. sativa* | Japonica^a^ | 30-00195 | China | 100 | Hap3 |
| Funingzipi | *O. sativa* | Japonica^a^ | 02-00058 | China | 99.6 | Hap4 |
| Baimaodao | *O. sativa* | Japonica^a^ | 07-00109 | China | 100 | Hap3 |
| Youmangzaojing | *O. sativa* | Japonica^a^ | 08-00253 | China | 100 | Hap3 |
| Heimangdao | *O. sativa* | Japonica^a^ | 28-00005 | China | 99.8 | Hap5 |
| Zhonglouyihao1 | *O. sativa* | Japonica^a^ | 04-00115 | China | 100 | Hap3 |
| Shuiyuan300li | *O. sativa* | Japonica^a^ | 02-00294 | China | 100 | Hap3 |
| LANI KHAMA | *O. sativa* | ARO^b^ | 26502 | Bangladesh | 99.5 | Hap2 |
| MADHABSAR | *O. sativa* | ARO^b^ | 34748 | Bangladesh | 99.4 | Hap6 |
| Dom Sofid | *O. sativa* | ARO^b^ | 17616 | Iran | 100 | Hap3 |
| Firooz | *O. sativa* | ARO^b^ | 10315 | Iran | 99.5 | Hap7 |
| JC1 | *O. sativa* | ARO^b^ | 68399 | India | 99.5 | Hap2 |
| DAL KATRA | *O. sativa* | ARO^b^ | 26451 | Bangladesh | 99.5 | Hap2 |
| Koshihikari | *O. sativa* | TEJ^b^ | 20526 | Japan | 100 | Hap3 |
| Beonjo | *O. sativa* | TEJ^b^ | 55457 | Korea | 100 | Hap3 |
| Mansaku | *O. sativa* | TEJ^b^ | 13338 | Japan | 100 | Hap3 |
| Tainan Iku 487 | *O. sativa* | TEJ^b^ | 9110 | Taiwan | 100 | Hap3 |
| Dholi Boro | *O. sativa* | TRJ^b^ | 13736 | Bangladesh | 99.4 | Hap6 |
| N 22 | *O. sativa* | TRJ^b^ | 25750 | India | 99.6 | Hap8 |
| Kotobuki Mochi | *O. sativa* | TRJ^b^ | 5474 | Japan | 100 | Hap3 |
| KU 115 | *O. sativa* | TRJ^b^ | 65907 | Thailand | 99.5 | Hap2 |
| Tebiezao | *O. sativa* | indica^a^ | 22-03030 | China | 99.5 | Hap2 |
| Suoyizhan | *O. sativa* | indica^a^ | 15-00636 | China | 99.5 | Hap2 |
| Qiyuexian | *O. sativa* | indica^a^ | 16-06887 | China | 99.5 | Hap2 |
| Haolai | *O. sativa* | indica^a^ | 21-02824 | China | 99.5 | Hap2 |
| Wenxiangnuo | *O. sativa* | indica^a^ | 21-00272 | China | 99.5 | Hap2 |
| Hongainuo | *O. sativa* | indica^a^ | 16-05252 | China | 99.5 | Hap2 |
| Sanbaili1 | *O. sativa* | indica^a^ | 18-02084 | China | 99.5 | Hap2 |
| Manliu | *O. sativa* | indica^a^ | 13-00449 | China | 99.5 | Hap2 |
| Feienuo2 | *O. sativa* | indica^a^ | 22-02356 | China | 100 | Hap3 |
| Shundetanghe | *O. sativa* | indica^a^ | 15-01270 | China | 99.5 | Hap2 |
| Qiuqianbai | *O. sativa* | indica^a^ | 11-00389 | China | 99.5 | Hap2 |
| Lucaihao | *O. sativa* | indica^a^ | 13-00816 | China | 99.5 | Hap2 |
| Sihuisanchaoqi | *O. sativa* | indica^a^ | 15-01465 | China | 99.5 | Hap2 |
| Zaoyouzhan | *O. sativa* | indica^a^ | 15-02740 | China | 99.5 | Hap2 |
| Liushizao | *O. sativa* | indica^a^ | 11-00322 | China | 99.5 | Hap2 |
| Sanbaili2 | *O. sativa* | indica^a^ | 12-01446 | China | 99.5 | Hap2 |
| Taichung Native 1 | *O. sativa* | indica^b^ | 7448 | Taiwan | 99.5 | Hap2 |
| Taducan | *O. sativa* | indica^b^ | 5215 | Phillipines | 99.5 | Hap2 |
| DV1 | *O. sativa* | AUS^b^ | 8803 | Bangladesh | 99.5 | Hap2 |
| Black Gora (Ncs12) | *O. sativa* | AUS^b^ | 25757 | India | 99.5 | Hap2 |
| T1 | *O. sativa* | AUS^b^ | 9479 | India | 99.5 | Hap2 |
| DV85 | *O. sativa* | AUS^b^ | 5207 | Bangladesh | 99.4 | Hap6 |
| ruf-KHM1 | *O. rufipogon* | perennial^b^ | 106332 | Cambodia | 99.5 | Hap2 |
| ruf-IND1 | *O. rufipogon* | perennial^b^ | 106458 | India | 99.9 | Hap14 |
| ruf-IND2 | *O. rufipogon* | perennial^b^ | 80506 | India | 99.6 | Hap21 |
| ruf-IND3 | *O. rufipogon* | perennial^b^ | 80542 | India | 99.5 | Hap16 |
| ruf-IND5 | *O. rufipogon* | perennial^b^ | 106110 | India | 99.6 | Hap18 |
| ruf-IND6 | *O. rufipogon* | perennial^b^ | 81861 | India | 99.2 | Hap24 |
| ruf-MMR | *O. rufipogon* | perennial^b^ | 80742 | Myanmar | 99.6 | Hap19 |
| ruf-NPL2 | *O. rufipogon* | perennial^b^ | 93219 | Nepal | 99.9 | Hap9 |
| ruf-CHN1 | *O. rufipogon* | perennial^a^ | DX01 | China | 100 | Hap3 |
| ruf-CHN2 | *O. rufipogon* | perennial^a^ | GZ04 | China | 99.9 | Hap11 |
| ruf-CHN3 | *O. rufipogon* | perennial^a^ | QY03 | China | 99.5 | Hap2 |
| ruf-CHN4 | *O. rufipogon* | perennial^a^ | TY01 | China | 99.5 | Hap17 |
| ruf-CHN5 | *O. rufipogon* | perennial^a^ | HN02 | China | 100 | Hap3 |
| ruf-CHN6 | *O. rufipogon* | perennial^a^ | HN1 | China | 100 | Hap3 |
| ruf-CHN7 | *O. rufipogon* | perennial^a^ | WN1 | China | 99.9 | Hap12 |
| ruf-CHN8 | *O. rufipogon* | perennial^a^ | WN2 | China | 99.9 | Hap10 |
| ruf-CHN9 | *O. rufipogon* | perennial^a^ | DX2 | China | 99.9 | Hap13 |
| ruf-CHN10 | *O. rufipogon* | perennial^a^ | GZ1 | China | 99.9 | Hap15 |
| ruf-CHN11 | *O. rufipogon* | perennial^a^ | QY1 | China | 99.5 | Hap2 |
| ruf-CHN12 | *O. rufipogon* | perennial^a^ | TY1 | China | 99.5 | Hap17 |
| ruf-CHN13 | *O. rufipogon* | perennial^a^ | BS2 | China | 99.5 | Hap17 |
| ruf-CHN14 | *O. rufipogon* | perennial^a^ | DX1 | China | 100 | Hap3 |
| niv-KHM | *O. nivara* | annual^b^ | 105724 | Cambodia | 99.6 | Hap20 |
| niv-IND2 | *O. nivara* | annual^b^ | 81861 | niv-IND2 | 99.6 | Hap22 |
| niv-IND3 | *O. nivara* | annual^b^ | 86474 | niv-IND3 | 99.6 | Hap22 |
| niv-IND4 | *O. nivara* | annual^b^ | 80559 | niv-IND4 | 99.5 | Hap7 |
| niv-IND5 | *O. nivara* | annual^b^ | 80558 | niv-IND5 | 99.5 | Hap7 |
| niv-IND6 | *O. nivara* | annual^b^ | 80545 | India | 99.2 | Hap25 |
| niv-MMR1 | *O. nivara* | annual^b^ | 80725 | niv-MMR1 | 99.6 | Hap23 |
| niv-MMR2 | *O. nivara* | annual^b^ | 80724 | niv-MMR2 | 99.5 | Hap2 |
| niv-NPL1 | *O. nivara* | annual^b^ | 105703 | niv-NPL1 | 99.8 | Hap22 |
| niv-NPL2 | *O. nivara* | annual^b^ | 105704 | niv-NPL2 | 99.8 | Hap22 |

^a^ indicates the germplasms from Chinese Crop Germplasm Bank.

^b^ indicates the germplasms from International Rice Research Institute.

^c^ indicates the sequence from NCBI.
