# Supplementary material for: Ehd4 Encodes a Novel and Oryza-Genus-Specific Regulator of Photoperiodic Flowering in Rice
Source: PLoS Genet. 2013 Feb 21;9(2):e1003281. doi: 10.1371/journal.pgen.1003281 (PMC3578780; doi:10.1371/journal.pgen.1003281)
Supplement: Table S3 — Primers used in this study. (DOC) [file pgen.1003281.s013.doc]

**Table S3.** Primers used in this study.

| **Primer name** | **Primer sequence (5'-3')** | | **Description** | |
| --- | --- | --- | --- | --- |
| Primers used for map-based cloning and marker-assisted selection | | | | |
| RM14282-F | CCCAAACACAAACACAAAGAGAGC | | SSR^a^ | |
| RM14282-R | AACACGCAGGTCCTCTTGAACC | |  | |
| RM14286-F | CAACAATGGCGTCGTTCTCTCC | | SSR | |
| RM14286-R | CGATTCGATTCTAGAGGGTTTAGGG | |  | |
| EJ-4-F | AACTTCCTCGTTCCCCATCT | | Indel^b^ | |
| EJ-4-R | CTTCTTCTTGGCTGCTTGCT | |  | |
| EJ-5-F | AGGACAGGTTTCAACCATCG | | Indel | |
| EJ-5-R | GAAGGGTGCGTTGGGTAGTA | |  | |
| RM6349-F | ATGATGCCTCATGTCTCTGATCTCC | | SSR | |
| RM6349-R | AGATGAACACGACCGATAGGATAGG | |  | |
| RM14302-F | TCATCATCATCAGTCCAGCATCG | | SSR | |
| RM14302-R | GCGGCGATTGAATTGTTTCTTAGG | |  | |
| RM14308-F | GCATTCTAGTGTTATGGCTGTTGC | | SSR | |
| RM14308-R | GCTACTAAGCTGGTCACCTGTGG | |  | |
| Ehd4-MAS-F | TGGAATCTCATCACGATCATTATCCTCCAT | | dCAPS^c^ | |
| Ehd4-MAS-F | AAGCAGTCCTGGACTCAGATGGACT | | NcoI (Enzyme) | |
| Primers used for transgenic construction | | | | |
| pEhd4-Ehd4-F | CCGGCGCGCCAAGCTTTTGCCGGTAGACACCAAATG | | | *pEhd4::Ehd4* construct complementation |
| pEhd4-Ehd4-R | GGGGATCCGTCGACCTGCAGTCAGGGGTGCAGGTAGTTACAAGATG | | |  |
| Ubi-Ehd4-F | TCTGCACTAGGTACCTGCAGATGCCGCAGACAAGTTCTAGTGCACA | | | *pUbi::Ehd4* construct |
| Ubi-Ehd4-R | GGGGATCCGTCGACCTGCAGTCAGGGGTGCAGGTAGTTACAAGATG | | |  |
| Ubi-Ehd1-F | TCTGCACTAGGTACCTGCAGATGGATCACCGAGAGCTGTGGCCTTAT | | | *pUbi::Ehd1 construct* |
| Ubi-Ehd1-R | GGGGATCCGTCGACCTGCAGTTAGAAATTCCAAAAACATGGTCCAT | | |  |
| pEhd4GUS-F | CCATGATTACGAATTCTTGCCGGTAGACACCAAATG | | | *pEhd4::GUS* construct |
| pEhd4GUS-R | CTCAGATCTACCATGGTGGATGGACTCCACAGTTCACTGTTGTG | | |  |
| Primers used for subcellular localization | | | | |
| Ehd4-PA7-F | TGGTCGACGTACTAGTGATGCCGCAGACAAGTTCTAGTGCACA | | | Ehd4-GFP fusion protein construct |
| Ehd4-PA7-R | GCTCACCATCACTAGTTAAGGGGTGCAGGTAGTTACAAGATGC | | |  |
| Primers used for transactivation activity assay | | | | |
| Ehd4-BD-F1 | CATGGAGGCCGAATTCATGCCGCAGACAAGTTCTAGTGCACACA | | pEhd4-1-832 | |
| Ehd4-BD-R1 | GGATCCCCGGGAATTCTTAGGGGTGCAGGTAGTTACAAGATGCA | |  | |
| Ehd4-BD-F1 | CATGGAGGCCGAATTCATGCCGCAGACAAGTTCTAGTGCACACA | | pEhd4-Δ806-832 | |
| Ehd4-BD-R2 | GGATCCCCGGGAATTCTTATTGTCCCCTTGGGGTTGATCTCGA | |  | |
| Ehd4-BD-F1 | CATGGAGGCCGAATTCATGCCGCAGACAAGTTCTAGTGCACACA | | pEhd4-Δ417-832 | |
| Ehd4-BD-R3 | GGATCCCCGGGAATTCTTAACTCTTAGATATCGGAGTTGGGC | |  | |
| Ehd4-BD-F2 | CATGGAGGCCGAATTCAAAGAGGTGTCTCCGCAATCTAAG | | pEhd4-417-805 | |
| Ehd4-BD-R2 | GGATCCCCGGGAATTCTTATTGTCCCCTTGGGGTTGATCTCGA | |  | |
| Primers used for prokaryotic expression | | | | |
| Ehd4-N-c2x-F | AAGGATTTCAGAATTCATGCCGCAGACAAGTTCTAG | | Ehd4-1-400 | |
| Ehd4-N-c2x-R | TAGAGGATCCGAATTCTTAACTCTTAGATATCGGAGTTG | |  | |
| Ehd4-C-c2x-F | AAGGATTTCAGAATTCAAAGAGGTGTCTCCGCAATCTAA | | Ehd4-401-832 | |
| Ehd4-C-c2x-R | TAGAGGATCCGAATTCTCAGGGGTGCAGGTAGTTACAAGATGC | |  | |
| Ehd4-C△-c2x-F | AAGGATTTCAGAATTCAAAGAGGTGTCTCCGCAATCTAA | | Ehd4-401-803 | |
| Ehd4-C△-c2x-R | TAGAGGATCCGAATTCTCATTGTCCCCTTGGGGTTGATCTCGA | |  | |
| Primers used for quantitative RT-PCR | | | | |
| Ehd4-F | | CAGCCAGCGGAATCATCAC | This study | |
| Ehd4-R | | CCAAATCCATCAGACCTACTCCT |  | |
| Ehd2-F | | CGACGACAATAGCTCGATCGC | 20 | |
| Ehd2-R | | GTGCATGGTCACGGAGCCTT |  | |
| Ehd3-F | | GACCACCTCGTCACCTACAAG | This study | |
| Ehd3-R | | GAGTGTCCCTCCAGCTAATCC |  | |
| Hd1-F | | TCAGCAACAGCATATCTTTCTCATCA | 20 | |
| Hd1-R | | TCTGGAATTTGGCATATCTATCACC |  | |
| Ehd1-F | | CCTACAGTGATTATGGCTTCA | This study | |
| Ehd1-R | | GTGCTGCCAAATGTTGCTC |  | |
| Hd3a-F | | GCTCACTATCATCATCCAGCATG | 20 | |
| Hd3a-R | | CCTTGCTCAGCTATTTAATTGCATAA |  | |
| RFT1-F | | TGACCTAGATTCAAAGTCTAATCCTT | 20 | |
| RFT1-R | | TGCCGGCCATGTCAAATTAATAAC |  | |
| OsMADS14-F | | TGGTGGAGAAGCAGAAAGTC | This study | |
| OsMADS14-R | | ATGAAGGAGGATGATGAGGAAC |  | |
| OsMADS15-F | | CCCTACCCTACAGGCTACATA | This study | |
| OsMADS15-R | | TAGGAAGCACTAGGTACGTGCTGA |  | |
| OsMADS50-F | | ATGCAATGACACCAAACCATC | This study | |
| OsMADS50-R | | GGTAGTGGAGTCTGCCGATC |  | |
| OsMADS51-F | | TCCTGTGCATAAGTTTGGCAGT | This study | |
| OsMADS51-R | | TCTACCTGGGATCAATCAGTGG |  | |
| DTH8-F | | CAGGAGTGCGTGTCGGAGTT | 30 | |
| DTH8-R | | GGTCGTCGCCGTTGATGGT |  | |
| Ghd7-F | | GCTTGAACCCAAACACGG | 30 | |
| Ghd7-R | | CTCATCTCGGCATAGGCTT |  | |
| UBQ-F | | ACCCTGGCTGACTACAACATC | This study | |
| UBQ-R | | AGTTGACAGCCCTAGGGTG |  | |
| LOC_Os08g23290-F | | AGTCTCCAGCACCTCAACATATCC | This study | |
| LOC_Os08g23290-R | | TCTGTCTATCCTTCCCTGAAACCG |  | |
| LOC_Os11g15340-F | | CGCCACCGCTACAACAGG | This study | |
| LOC_Os11g15340-R | | GCTCTCCTCCCACGCACAG |  | |
| LOC_Os07g41370-F | | ATCCCATCCCGACAACAAAT | This study | |
| LOC_Os07g41370-R | | GCTTGCTGTTGCCTGCTTGT |  | |
| LOC_Os03g08460-F | | GTGCAGCAGCCTATGCAGAT | This study | |
| LOC_Os03g08460-R | | GCGTCCTCCTCCATGAGC |  | |

^a^ Simple Sequence Repeat marker (Microsatellite marker).

^b^ Insertion-deletion sequence marker.

^c^ Cleaved amplified polymorphic sequence marker.

Red letter represents recombination tag.
